# MXene-bioinspired nanomaterials for biosensing of pharmaceuticals and personal care products in complex aquatic systems at ultra-trace levels

**DOI:** 10.1186/s11671-026-05013-7

**Published:** 2026-10-26

**Authors:** Seyed Morteza Naghib, Mohammad Ali Khorasani, Fariborz Sharifianjazi, Ketevan Tavamaishvili

**Affiliations:** 1 Nanotechnology Department, School of Advanced Technologies, University of Science and Technology (IUST), Tehran, 1684613114 Iran; 2 Biomaterials and Tissue Engineering Research Group, Interdisciplinary Technologies Department, Breast Cancer Research Center, Motamed Cancer Institute, ACECR, Tehran, Iran; 3 Center for Advanced Materials and Structures, School of Science and Technology, The University of Georgia, Tbilisi, 0171 Georgia; 4 School of Medicine, Georgian American University, 10 Merab Aleksidze Str, 0160 Tbilisi, Georgia

**Keywords:** MXene, Electrochemical biosensors, Bio-inspired platforms, Pharmaceutical detection, Personal care products, Complex aquatic systems

## Abstract

Pharmaceuticals and personal care products (PPCPs) are an increasing class of environmental pollutants of concern in aquatic ecosystems owing to their persistence and biological activity at low concentrations. Correct and selective monitoring of these substances is necessary to assess their ecological impacts and preserve water quality. The high conductivity, large surface area and tunable surface chemistry of two-dimensional transition metal carbides and nitrides (MXenes) have recently opened new avenues for electrochemical sensing. In this study we explain the state of the art of MXene nanoparticles, their characteristics, production and functionalization procedures that allow the integration of these materials in electrochemical and bioinspired sensing systems. Design advances such as biomimetic recognition elements, molecular imprinting, enzymes and nanocomposites are critically reviewed in terms of their contribution towards improving selectivity and operational stability of sensors. This study examines the analytical performance of MXene-based sensors for the ultra-trace detection of PPCPs in complex aquatic matrices, focusing on advances in sensitivity, antifouling strategies, and multiplexed detection. It also discusses future perspectives such as smart sensor networks and AI-driven data analytics, as well as issues in material scalability, sensor durability and real-world deployment. This study opens the door for the new potential of MXene-enabled platforms for the development of environmental analytical chemistry and shows the future research pathways towards sustainable and effective aquatic pollution monitoring.

## Introduction

**Fig. 1:**
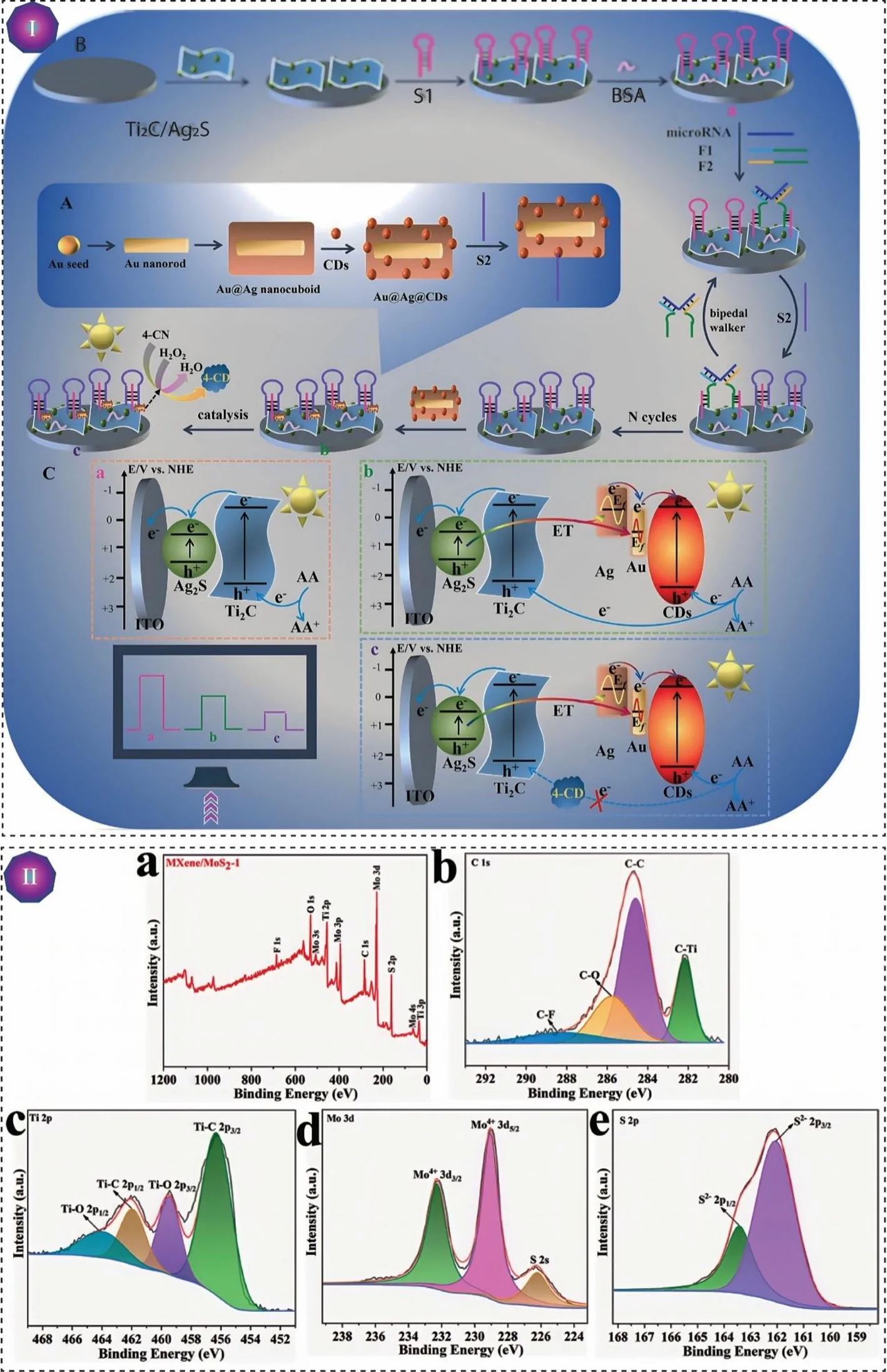
(I) Photoelectrochemical biosensor for the identification of miRNA-141 [16]. (II) (**a**) XPS survey; (**b**) C 1s, (**c**) Ti 2p, (**d**) Mo 3d, and (**e**) S 2p of MXene/MoS2-1 [15]

PPCPs are a complex and diverse group of chemical contaminants, which have gained significant global interest for their ubiquitous occurrence and persistence in the aquatic environment [1–3]. This category includes prescription and non-prescription medications, antibiotics, analgesics, hormones, perfumes and many popular personal care and cosmetic goods, which are rapidly being recognized as novel contaminants in surface and groundwater ecosystems [4–6]. Recent developments in analytical technology have made it possible to identify PPCPs in a wide range of environmental matrices (water, sediments and biota) at concentrations ranging from ng L^− 1^ to µg L^− 1^ [7–9]. The constant input and frequent detection of PPCPs in aquatic systems is mainly owing to the continuous release of PPCPs from urban, industrial and agricultural sources, with wastewater treatment plants being the key conduits for their introduction into the environment [10–12]. Conventional approaches for wastewater treatment are not enough to completely remove PPCPs, and residual and transformation products are discharged into receiving waters [13, 14]. PPCPs released into the aquatic environment may bioaccumulate in aquatic organisms, particularly invertebrates, and enter food webs, which may cause acute and chronic ecotoxicity [15]. Figure 1 shows thoroughly investigated structural and chemical characteristics of the MXene/MoS_2_ composites.

**Fig. 2:**
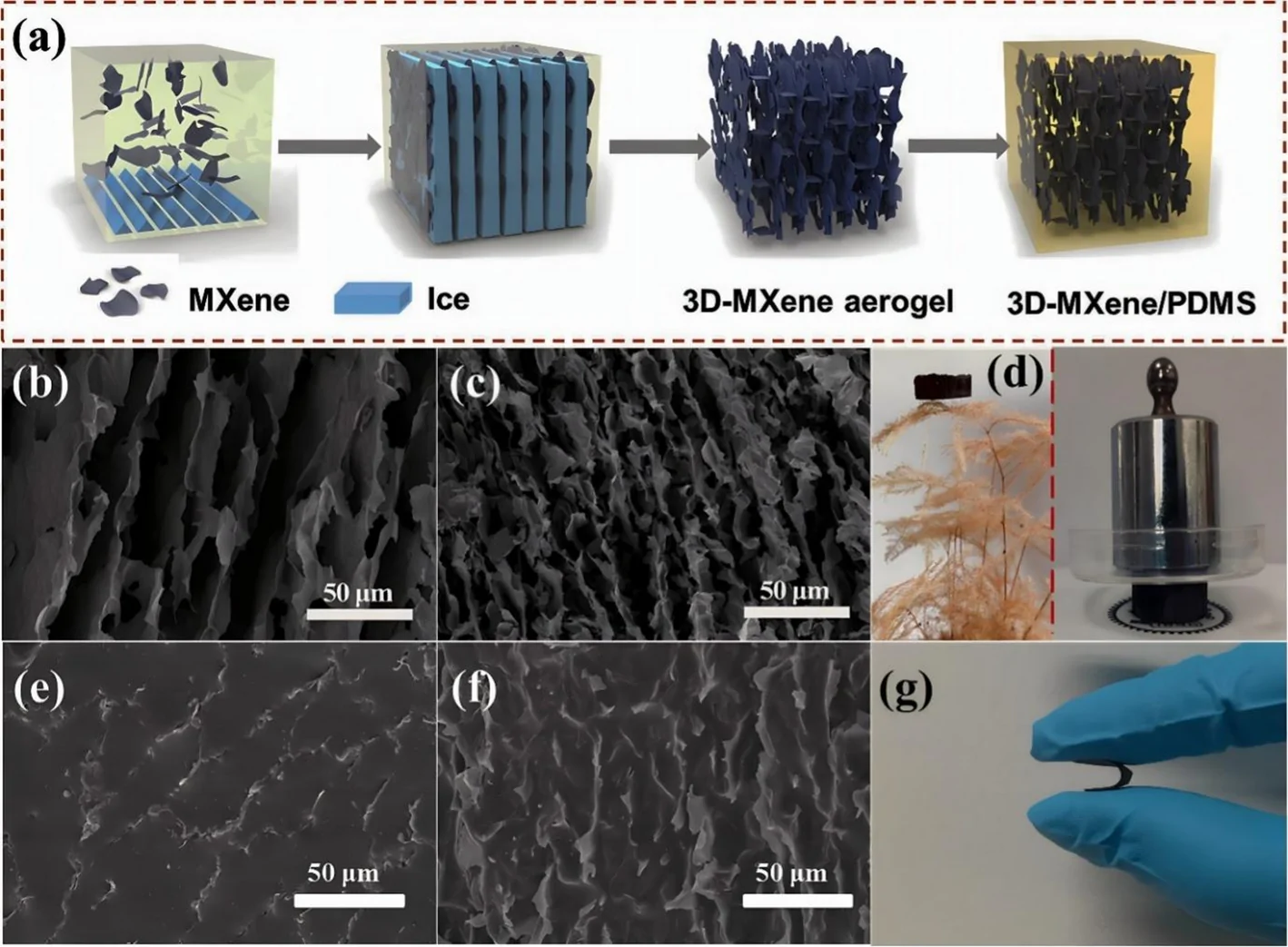
Fabrication and structural properties of 3D-MXene aerogels: (**a**) schematic illustration of the ice-templating and polymer embedding process from MXene nanosheets to 3D-MXene/PDMS composite. (**b**, **c**, **e**, **f**) SEM images showing porous and interconnected 3D architectures. (**d**, **g**) digital photographs demonstrating the lightweight and flexible nature of the freestanding aerogel composites [38]

Although PPCPs are ubiquitous at trace levels, there is increasing worry about their potential to have sublethal physiological, reproductive and behavioral effects [17–19]. Many are endocrine-disrupting compounds (EDCs) that may interfere with hormone control in fish, amphibians and other animals. These results are especially important since several PPCPs such as synthetic estrogens and selective serotonin reuptake inhibitors exhibit considerable chronic toxicity to aquatic organisms at ecologically relevant amounts [20–22]. The rising knowledge of the many risks associated with PPCPs in aquatic systems has led to a greater effort on the part of regulatory agencies and the scientific community to conduct occurrence monitoring and ecotoxicological studies [23–26]. They are also searching for more efficient ways to assess and correct. The considerable chemical diversity, variable permanence and possibly synergistic effects of PPCP chemicals provide a great challenge to the preservation of aquatic ecosystems, making risk assessment and water quality management approaches difficult to use [27–29]. Thus, ultra-trace detection of PPCPs and development of new selective and sensitive monitoring approaches are key priorities in environmental analytical chemistry [30–32]. Recently, innovative material systems such as MXenes, two-dimensional transition metal carbides and nitrides, have emerged as potential solutions in this regard for the creation of next-generation electrochemical and bio-inspired sensors [33, 34]. They provide unique prospects to enhance the identification and protection of PPCPs in aquatic systems dramatically [35, 36]. The shape, content and fabrication of the 3D-MXene aerogels are shown in Fig. 2. SEM photos of various diameters show porous lamellar structures, which are corroborated by EDX spectra and elemental mapping of Ti, N and S. The schematic describes the ice-templating and polymer embedding process for the fabrication of 3D-MXene/PDMS composites, and their light, flexible, and interconnected structures are shown in the SEM and digital photos [37].

While various recent reviews have highlighted the rapid growth of MXenes in sensing applications, there are still considerable shortcomings in their use in PPCPs in complex aquatic settings. The existing literature mainly covers MXene-based electrochemical sensing as a comprehensive analytical framework, focusing on sensor development, electrochemical processes, and general categories of analytes. The reviews on environmental sensing include a broader range of pollutants such as gases, heavy metals, pesticides, and organic contaminants, but they do not specifically address PPCP detection in real aquatic environments. Recent investigations on MXene biosensors have been mostly focused on biomarker detection, clinical diagnostics, wearable and biological recognition, and not on a comprehensive investigation of pharmaceutical and personal-care contaminants in ambient water sources. Recent dedicated evaluations of MXenes in pharmaceutical analysis have started to focus on drug detection. However, they mainly focus on electrochemical pharmaceutical analysis and sensor efficacy instead of the larger environmental issue of ultra-trace PPCP surveillance in complex aquatic environments. Thus, this review is distinguished by the integration of previously disparate perspectives into a PPCP-centric framework, focusing on bio-inspired recognition, surface functionalization, antifouling approaches, matrix effects, ultra-trace analytical capacity, multiplex detection and the shift from regulated laboratory analyses to the monitoring of real aquatic ecosystems. Such integration adds another dimension to the present MXene sensing literature and solves the analytical and practical challenges of detecting PPCPs at ultra-trace levels in complex aquatic environments.

## Synthesis, characteristics and applications of nanomaterials of MXene

**Fig. 3:**
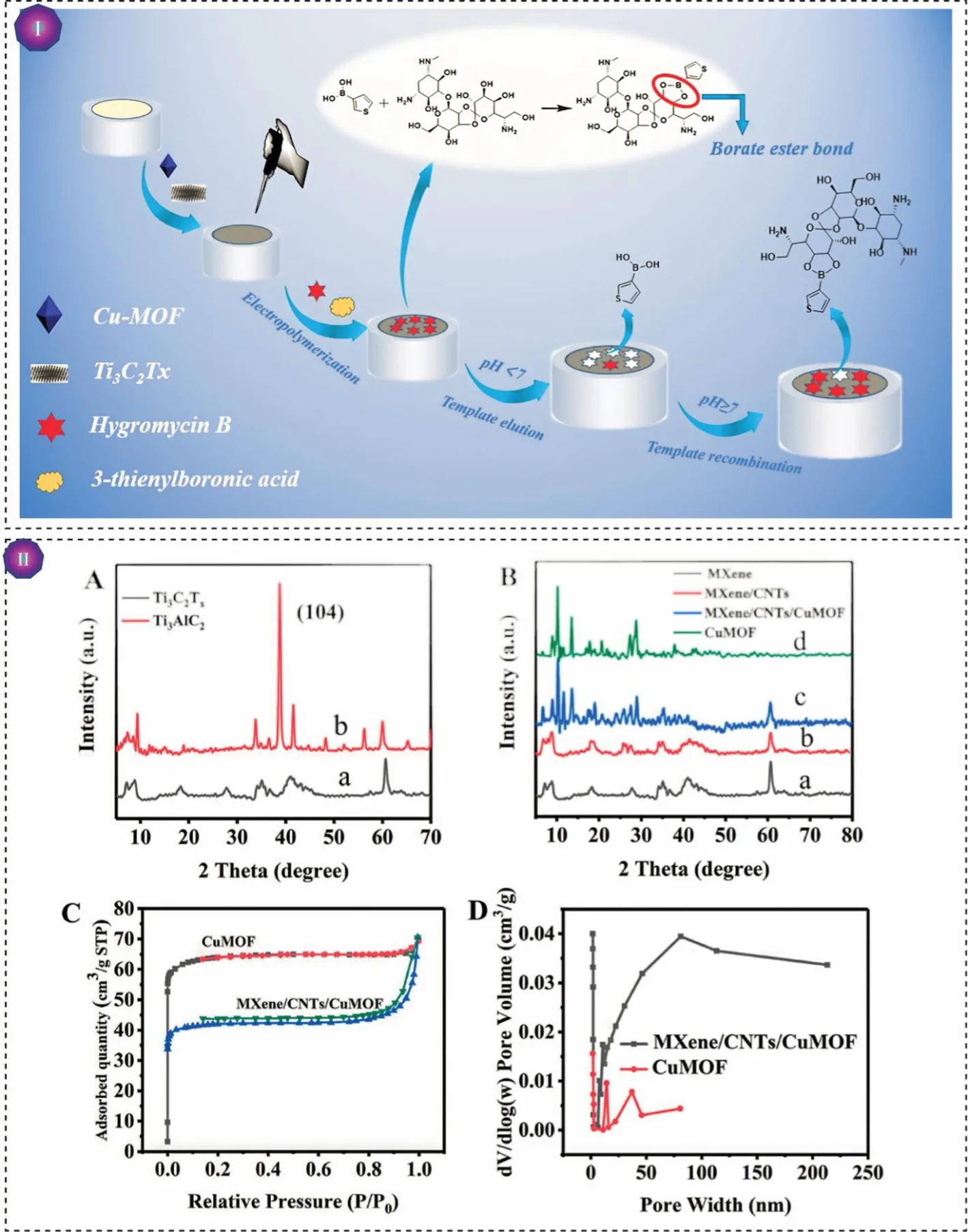
(I) Schematic preparation of MIP/Cu-MOF/Ti_3_C_2_T_x_/GE and electrochemical detection of hygromycin B [50]. (II) XRD of (**A**) Ti_3_C_2_T_x_ (a) and Ti_3_AlC_2_ (b); XRD of (**B**) MXene (a), MXene/CNTs (b), MXene/CNTs/CuMOF (c) and CuMOF (d). (**C**) Nitrogen adsorption and desorption isotherms measured at 77 K. (**D**) Pore-size distribution. EDS analysis of CuMOF (E) and MXene/CNTs/CuMOF [51]

In addition to these intrinsic properties, the structural tunability of MXenes might lead to ultra-trace detection limits of PPCPs in aquatic environments [5, 39]. Several high-impact research studies published in the years between 2023 and 2025 have revealed that functionalized Ti_3_C_2_T_x_ and Nb_2_CT_x_ MXenes may reach very low detection limits for non-steroidal anti-inflammatory medications, antidepressants and UV filters. Researchers have shown that these MXenes have significantly enhanced performance compared to graphene and MoS_2_ based nanosensors. The latter are usually stated to have detection limits less than 10^− 10^ M under the identical testing circumstances [16]. This quantitative advantage arises from the unique combination of electronic conductivity of over 10,000 S cm^− 1^ in delaminated pristine Ti_3_C_2_T_x_ films and high density of hydrophilic terminations allowing strong adsorption and fast charge-transfer kinetics with bio-relevant metabolites and pharmaceutical residues [17]. Table 1 shows the comparison of structural, electrical and functional properties of MXenes with common 2D nanomaterials. This balance is essential to provide excellent sensing performance. In a analysis of several peer-reviewed studies of MXene-based sensors published since 2023, the average increase in high sensitivity and specificity was shown in comparison to sophisticated carbon-based electrochemical biosensors [40]. These advances show the translational supremacy of MXenes in complex water matrices. Methods for functionalization have been developed [41–43]. Surface oxidation in aquatic conditions was a challenge for prior-generation MXene electrodes. Redox-stable coatings such as polydopamine and zwitterionic polymers increase structural integrity and improve selectivity for structurally comparable medicinal metabolites [44]. In one such work, Ti_3_C_2_T_x_-polydopamine composites were used to detect carbamazepine in wastewater effluent, with low detection limits. This performance is beyond the limit of detection of liquid chromatography-mass spectrometry (LC-MS) standards. Importantly, the in situ performance test at the hospital wastewater treatment facilities showed excellent recovery rates in the absence of any additional preconcentration steps [45, 46]. Several works provide a foundation for understanding MXene robustness. From a clinical translational approach, the functionalized MXene systems have been included in environmental monitoring and large-scale public health surveillance [47–49] (Fig. 3).

**Table 1:** Structural and functional characteristics of MXenes versus conventional 2D nanomaterials for electrochemical sensing applications

Material System	Electrical Conductivity (S cm⁻¹)	Specific Surface Area (m² g⁻¹)	Surface Terminations / Functional Groups	Typical LOD for PPCPs*	Environmental Stability	Key Advantages	Key Limitations	References
Double-Network Hydrogel with Continuous Ti₃C₂Tₓ MXene Nanochannels (PcAT MNC)	Pure MXene: 1700 S cm⁻¹ (1.7×10⁵ S/m)• Hydrogel: 0.094 S cm⁻¹ (9.4 S/m)	High internal surface area from 3D continuous hollow nanochannel networks	Terminal groups: O 1s, F 1s, Ti 2p, C 1s, plus phenolic -OH from Tannic Acid	N/A(Piezoresistive/capacitive sensor; Pressure LOD: 213 Pa)	• Anti-freezing at -18°C for 12 h• Anti-dehydration (52% weight retention at 37°C for 14 days)	• High pressure sensitivity (4.98 kPa⁻¹) & low threshold (213 Pa)• Tissue self-adhesion (25 kPa) & extreme stretchability (1400%)	• Multi-step template synthesis (electrospinning, UV crosslinking, organic solvent leaching)• Requires extensive washing	[44]
Photoactive Porous MXenes (Ti₃C₂) & Semiconductor Heterojunctions (e.g., g-C₃N₄/Ti₃C₂, BiVO₄/Ti₃C₂, AgBr/Ti₃C₂)	Metallic conductivity(Acts as electron sink and co-catalyst)	High specific surface area from porous 2D nanolayers	-F, -OH, -O terminations and exposed connector Ti metal sites	• Ciprofloxacin: 0.13 nM• Oxytetracycline: 0.03 nM• Streptomycin: 0.3 nM• Chlorpyrifos: 0.33 pg/L	Schottky barrier effectively suppresses charge recombination and electron backflow	• Femtomolar to nanomolar ultra-low detection limits• Low background noise via decoupled light excitation & electrical readout	• Relies on target acting as electron donor• Surface fouling from organic species• Lack of commercial integrated PEC devices	[16]
Monolayer Ti₃C₂ Nanosheets loaded with Gold Nanoparticles (AuNPs/Ti₃C₂) as SPRi conjugates	High metallic conductivity of monolayer Ti₃C₂(Plasmonic substrate)	Large specific surface area for high-density AuNP immobilization	-F, -OH, -O, functionalized with PolyA-DNA capture probes	N/A(Targeted for cancer biomarker miRNA-21; LOD = 6.13 fM)	High single-base mismatch selectivity and stable probe immobilization	• Femtomolar ultrasensitivity (1000× lower LOD than conventional DNA-AuNPs)• Dual mass & LSPR electromagnetic signal amplification	• Requires expensive specialized SPRi optical equipment• Complex multi-step hybridization procedure	[33]
Dip-coated Ti₃C₂Tₓ MXene / Waterborne Polyurethane (WPU) conductive shell on Polyurethane (PU) elastic fiber	Not explicitly stated in S cm⁻¹(Piezoresistive strain sensor)	High specific surface area from 2D MXene sheets (exact value N/A)	Hydrophilic polar groups:-OH, -F, -O (MXene) and hydrophilic WPU chains	N/A(Engineered for physical strain & human motion; LOD: 0.5% strain)	• <10% ΔR/R₀ after 1 h ultrasonication in water, saline, detergent, & tape peeling• Durable over >2500 stretch-release cycles	• Ultra-high strain sensitivity (Gauge Factor ≈ 960.8 at 65–90% strain)• Broad working range (0–90%)• High wash and wear durability	• Lacks chemical/biological target selectivity• High MXene loading reduces maximum stretchability	[5]
Surface-Engineered MXenes & MXene Composites (e.g., Ti3C2Tx, Nb2C, Ta2C, Ti4N3Tx, and polymer nanocomposites)	Metallic conductivity across 2D sheets; provides high electrical conductivity and rapid charge transfer in composite architectures	High specific surface area with versatile geometrical configurations (2D single/few-layer sheets, crumpled nano-coatings, 3D porous networks)	Primary: -F, -O, -OH; extended: -Cl, -Br, -I, -S, -Se, -Te, -NH (-OH offers highest binding energy)	High sensitivity across environmental and biological sensing (gas sensors, biosensors, heavy metal adsorption, and biomolecule tracking)	Aqueous stability, high structural/mechanical robustness, thermal stability, and corrosion/oxidation resistance via surface functionalization	• Combined metallic conductivity, high mechanical flexibility, hydrophilicity, biocompatibility, and highly tunable optical/electronic properties	• Hazardous HF-based etching, oxidation risk during ambient/alkali processing, defect creation during prolonged sonication/etching, and commercial scale-up challenges	[45]
3D Melamine-doped rGO / Ti₃C₂Tₓ MXene composite aerogel (3D MGMA) on Screen-Printed Carbon Electrode	High bulk network conductivity(Electroactive area A = 0.21 cm², 3.5× bare electrode)	High surface area from 3D interconnected honeycomb porous structure	-NH₂ (amino groups with lone pairs), -F, -O, -OH, and C-Ti-OH	N/A(Targeted for heavy metals: Zn²⁺: 0.48 µg/L, Cd²⁺: 0.45 µg/L, Pb²⁺: 0.29 µg/L)	• RSD < 0.82% across 10 sensing cycles• High anti-interference against 10-fold non-target ions	• Simultaneous multi-element heavy metal detection at ppb levels• Dense amino complexation sites• Low charge transfer resistance	• Requires wet digestion pre-treatment for food grain samples• Multi-step aerogel self-assembly	[10]

**Fig. 4:**
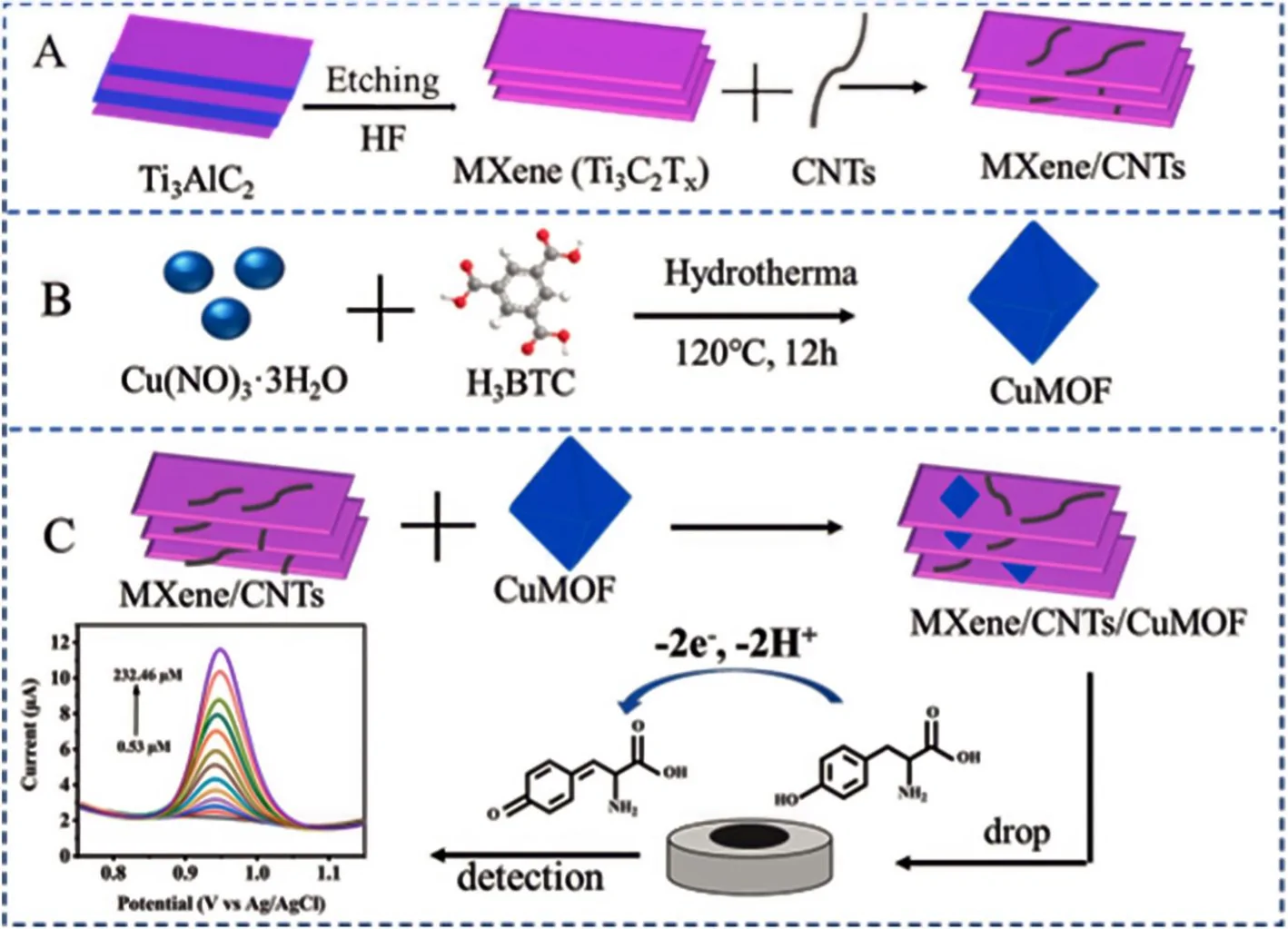
(**A**) Synthetic route for integrating Ti₃C₂Tₓ MXene, CNTs, and (**B**) Cu-MOF, followed by (**C**) modification for electrochemical detection [51]

Recent wastewater-based biosensors has shown that MXene-based electrochemical sensors can accurately predict and quantify antidepressants such as sertraline and fluoxetine in urban sewage. Reference [52] observed favorable correlation values. The trial conducted at several locations shows that MXene devices are not only laboratory curiosities but also possibly real-time epidemiological monitors. This enables linkage of patterns of pharmaceutical exposure with public health data which cannot be obtained with standard chromatographic approaches owing to increased infrastructure requirements [53]. The experimental findings are in agreement with the logic of MXene-based biosensing devices as a tool for scaled monitoring (Fig. 4).

**Fig. 5:**
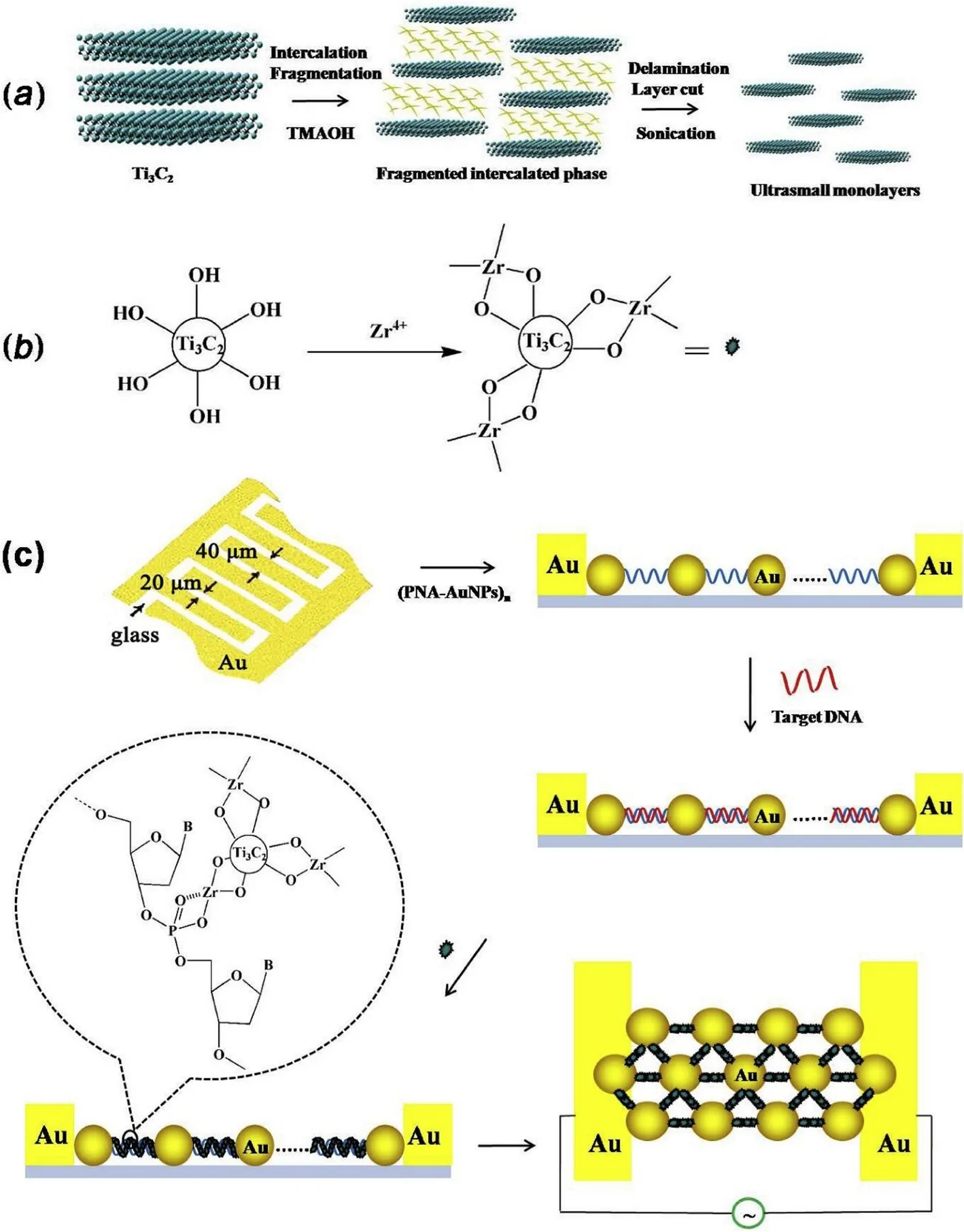
(**a**) The preparation of Ti3C2 MXenes nanosheets. (**b**) The pretreatment of Ti3C2 MXenes nanosheets with ZrOCl2. (**c**) The constructed strategy of sensor for rapid detection of M. tuberculosis [60]

**Fig. 6:**
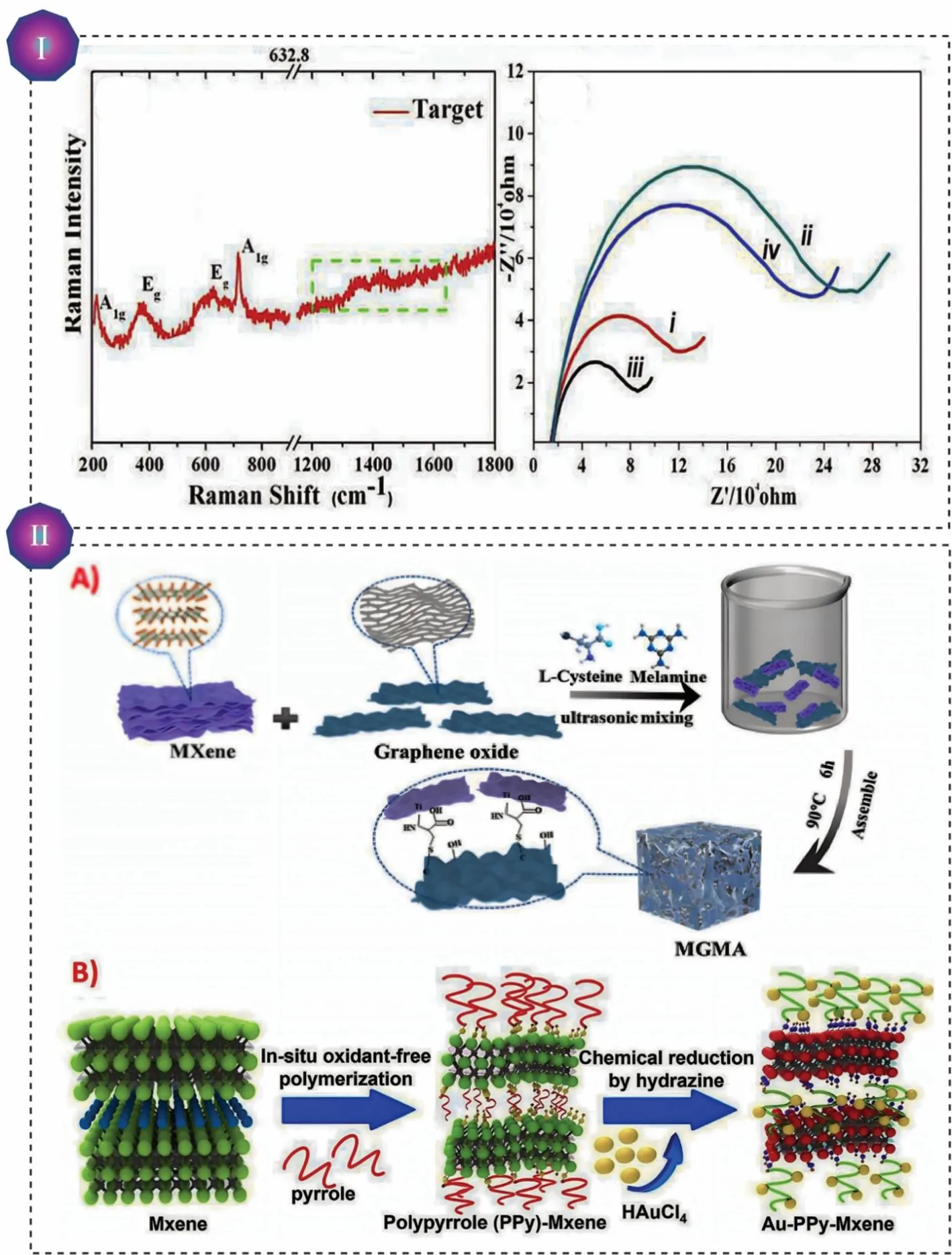
(I) Raman spectra, and electrochemical impedance results for detection performance analysis [60]. (II) (**A**) Schematic diagram of the fabrication process of 3D MGMA [10]. (**B**) Schematic of the preparation of Au-PPy-MXene-GCEs [17]

**Fig. 7:**
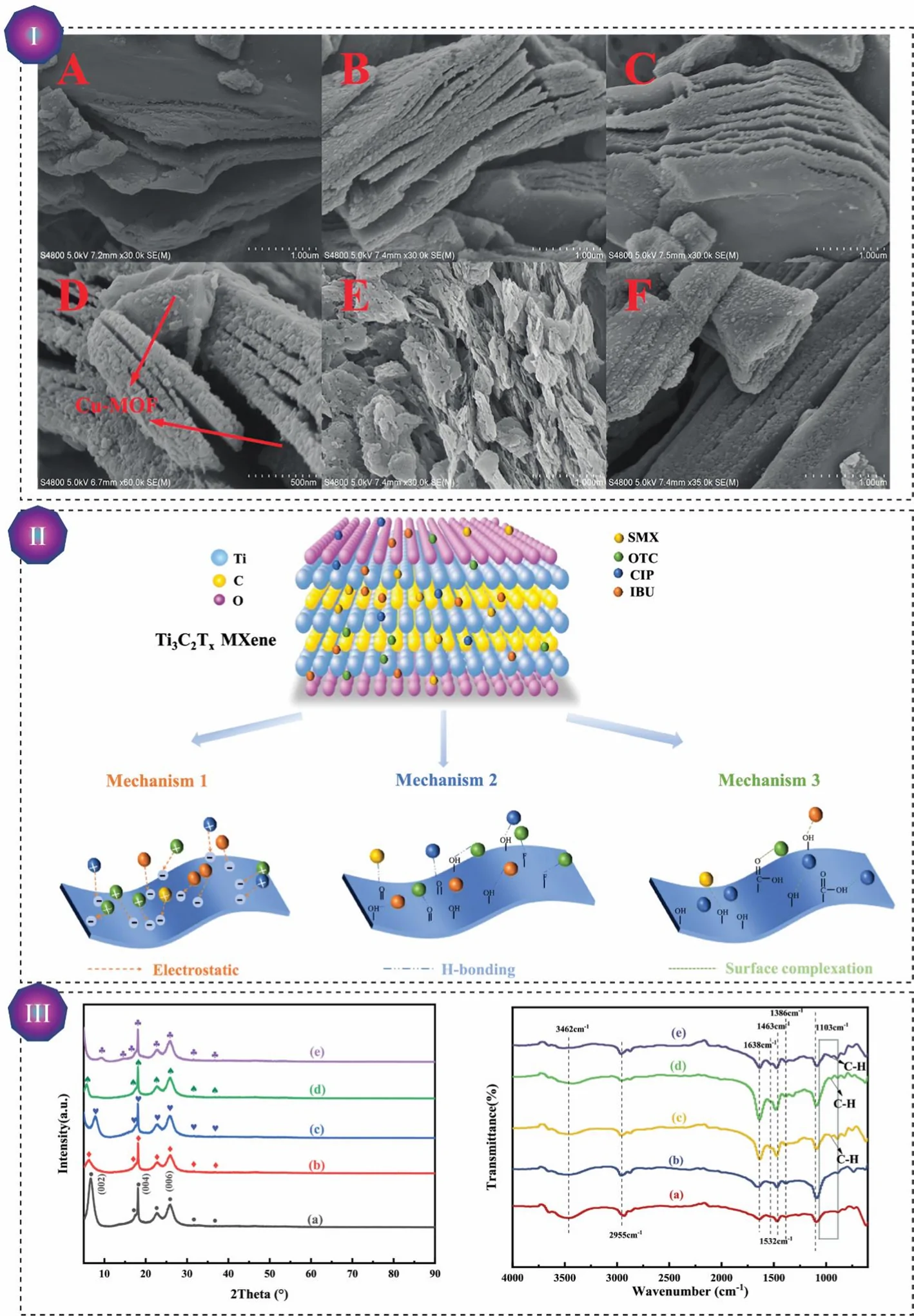
(I) SEM micrographs showing layered and porous morphologies of Cu-MOF and MXene/CNTs/Cu-MOF composites [50]. (II) Possible mechanisms of four PPCPs adsorption by Ti3C2Tx MXene [61]. (III) The FT-IR spectrum of Ti3C2Tx before and after use. (before use (a), after adsorption in surface water without PPCPs (b), after adsorption of 4 PPCPs in simulated solution (c), after adsorption of 4 PPCPs in surface water (d), after adsorption of CIP in simulated solution (e)) [61]

Emerging bio-inspired strategies have broadened the creative potential of MXene-based sensors [54]. Peptide-functionalized Ti_3_C_2_T_x_ nanosheets resembling fish olfactory receptor proteins demonstrated sub-picomolar selectivity to musk compounds contained in personal care items [55–57]. Researchers showed that the nanosheets boost the sensitivity of enzyme-based biosensors and decrease cross-reactivity [58]. The direct bio-mimetic molecular imprinting on MXene substrates has produced extremely reproducible recognition sites with an excellent intra-batch variability. This is an improvement compared to typical impressions of conducting polymers. These bio-inspired approaches improve biosensor repeatability, a critical barrier for regulatory approval and field deployment, as well as analytical performance [59]. Figure 5 shows a schematic illustration of the synthesis, structural characterization and electrochemical biosensing applications of MXene/CNTs/Cu-MOF nanocomposites (Figs. 6 and 7).

**Fig. 8:**
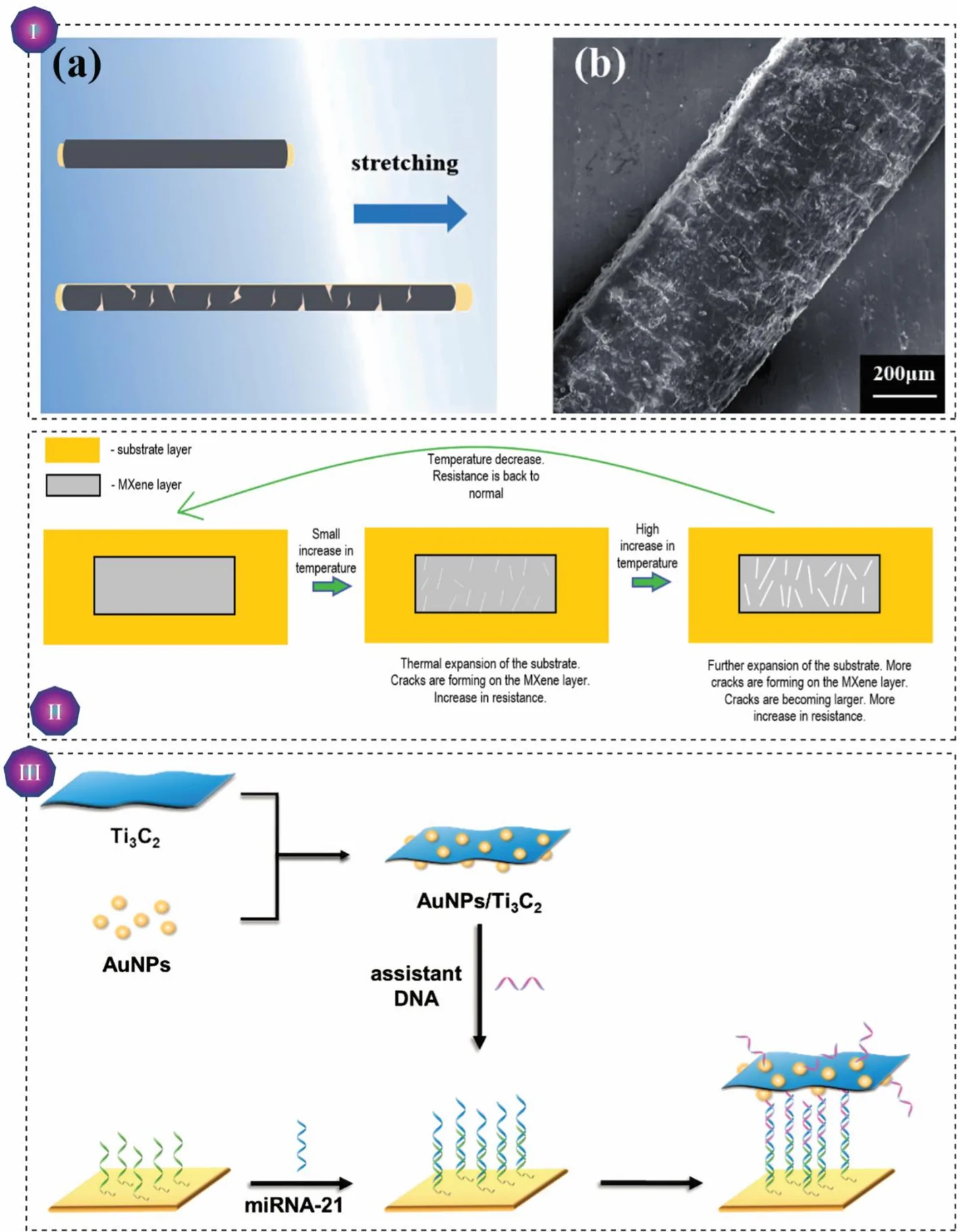
(I) Stretchable device structure: optical image (**a**) and SEM micrograph (**b**) of stretchable MXene-based film [5]. (II) Schematic fabrication of flexible sensors via Ti₃C₂ hybrid integration for enhanced conductivity and mechanical compliance [27]. (III) Schematic of the detection process of AuNPs/Ti3C2-SPRi biosensor [33]

MXene composites containing enzyme nanoclusters and catalytic metals have been used for hybrid electrochemical-bio-inspired sensing at the translational systems level [62, 63]. Horseradish peroxidase-AuNPs functionalized Ti_3_C_2_T_x_ sensors show superior performance than commercial electrochemical enzyme sensors, with detection ranges from femtomolar to micromolar concentrations [64]. Researchers found that R^2^ correlation values were mostly over 0.99 for many pharmacological classes. For the response time for the practical wastewater biosensor, it was 4 to 5 s when we evaluated it, but the response time was three times and faster than 1.5 s. The parameters greatly boost the mobility and scalability of actual applications. A major advance is the hybrid platforms that combine bio-inspiration recognition techniques with the high conductivity and configurable interface chemistry of MXenes [65]. These allow for ultrasensitive, selective and real-time detection of PPCPs in complicated aquatic environments [66]. Finally, MXene nanoparticles have the highest structural flexibility, complex functionalization, and bio-inspired recognition for PPCP sensing technology [67]. Quantitatively superior to the present nanomaterials and validated in large-scale epidemiological studies. Multifunctionally adaptable, hence a translationally possible, theoretically unique technology [68]. This is a major shift in ambient biosensing in terms of the combination of ultra-trace detection capabilities, robustness in complex aquatic matrices and potential for therapeutic application [69]. MXenes are expected to be disruptive platforms in the near future spanning environmental research, clinical diagnostics and public health monitoring [70]. Figure 8 shows Ti_3_C_2_-based nanohybrids for gas, pressure and strain sensing: Structural design, adsorption mechanism and multifunctional applications.

## MXene-based electrochemical sensors for detection of PPCPs

In this portion, the sensing investigations are assessed based on their application matrix and goal to avoid the misinterpretation of analytical performance in intrinsically different sample settings. The focus is on the determination of PPCPs in environmental and aquatic matrices, including wastewater, surface water and other complex water samples. Studies in biological matrices such as blood and interstitial fluid are considered separately as the evidence of the broad biosensing capabilities of MXene surfaces and are not directly compared with ambient PPCP sensors. Results obtained in buffer and other controlled laboratory settings may also be distinguished from tests performed on actual material. Thus, detection limits, specificity, robustness, recovery and time to result are interpreted within the framework of the relevant matrix, rather than as directly comparable performance indicators across environmental, clinical and laboratory settings.

**Fig. 9:**
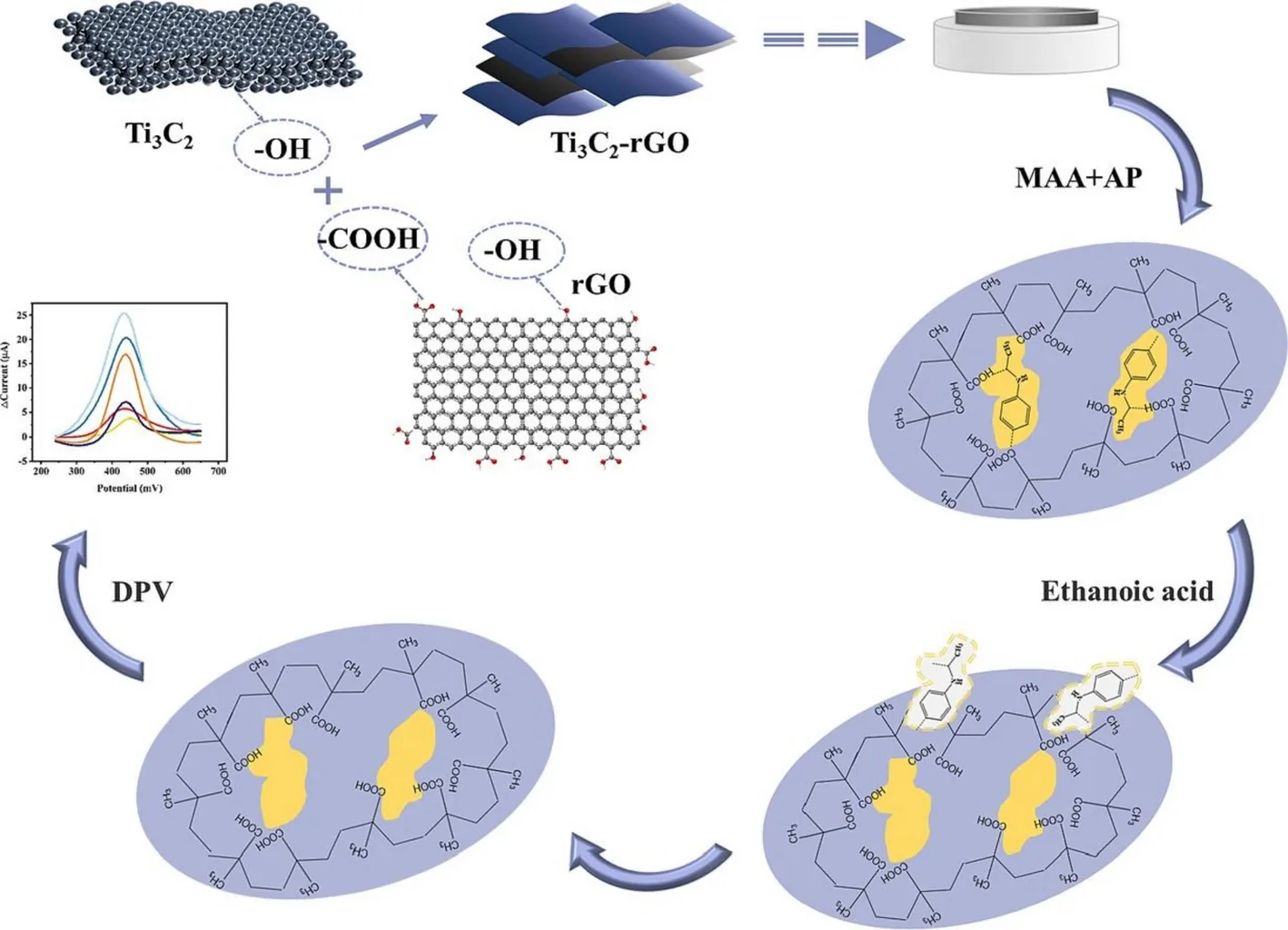
Schematic illustration of the fabrication and sensing mechanism of the molecularly imprinted electrochemical sensor based on rGO/Ti₃C₂Tₓ for selective acetaminophen determination [82]

Recent developments in the surface functionalization techniques [27] for MXene-based electrochemical sensors have paved the way for the detection of pharmaceuticals and personal care products. The outcomes of performance and clinical validation for these techniques are far better than those of the standard modification procedures. Recent investigations showed that the specific functionalization of the surface of Ti_3_C_2_T_x_ MXene with recognition elements along with aptamer results in excellent detection limits in the very low concentration range. Functionalized Ti_₃_C_₂_T_x_ MXene electrodes may be used as better electrochemical sensors with aptamers, enzymes, molecularly imprinted polymers and other recognition elements in many recent investigations [71–74]. However, the analytical efficiency of these platforms should be examined in relation to the sample matrix and the intended purpose. MXene-based interfaces have been investigated for environmental applications such as the detection of pharmaceutical contaminants and PPCPs in wastewater and various aquatic environments, where the performance of the sensor can be greatly influenced by matrix-derived interferents, natural organic constituents, competing ions and fouling processes. Conversely, studies conducted in biological matrices, such as human blood and interstitial fluid, primarily focus on biomedical biomarker detection and therapeutic drug monitoring rather than the detection of PPCPs in ambient water. Thus, while their analytical characteristics are not directly compared with those obtained from ambient water samples, these clinical and bioanalytical studies are viewed as another demonstration of the flexibility of MXene-based recognition interfaces. This distinction is important, since matrix composition, target concentration, sample preparation, and validation requirements are quite different for environmental vs. biological sensing applications. Clinical validation experiments from 2023 to 2025 showed excellent sensitivity and specificity of MXene-enhanced aptasensors to discriminate between positive and negative pharmacological biomarker samples in human blood matrices. The receiver operating characteristic (ROC) curves and cut-off analysis [75–77] statistically validated the diagnostic accuracy. The use of noble metal nanoparticles on MXene substrates is also a breakthrough for the signal amplification methods for ultra-trace medicine detection [78]. Synergistic electrocatalytic platforms may be generated by the in-situ formation of gold and platinum nanoparticles on the surface of Ti_3_C_2_T_x_ which can enhance the redox signal compared to pure MXene electrodes [79–81]. The hybrid nanoarchitectures show excellent analytical capabilities with detection limits down to the picomolar concentration level and linear detection ranges spanning over six orders of magnitude (Fig. 9).

**Fig. 10:**
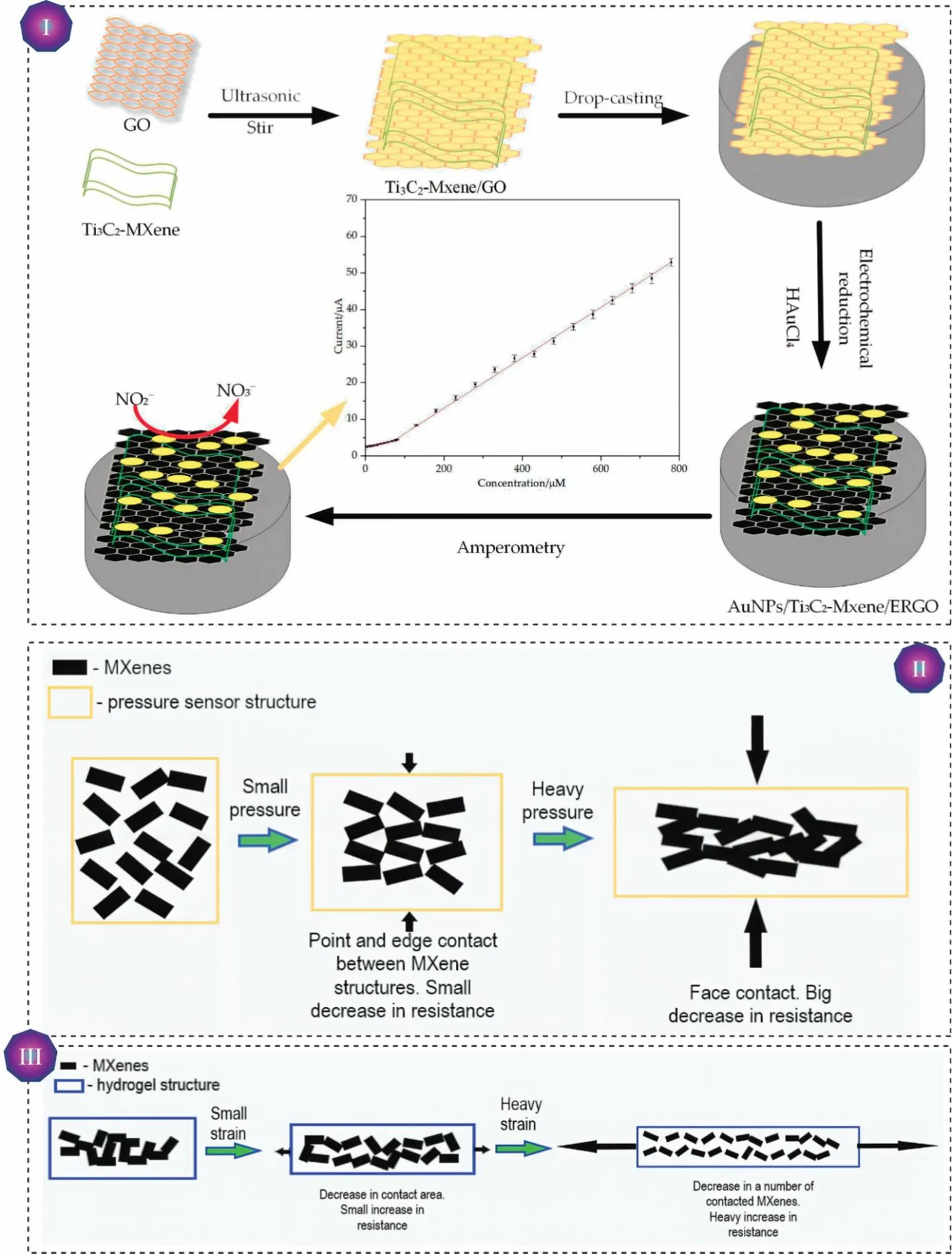
(I) Fabrication route of Ti₃C₂/MXene-GO hybrid with AuNPs via ultrasonic-assisted dispersion and drop-casting, with amperometric response to NO₂ gas [90]. (II) Pressure sensor structure: variation of contact area under low and high pressure and its effect on resistance [27]. (III) Strain sensing mechanism: conductive pathway rearrangement during stretching and releasing cycles [27]

Multi-metal nanocomposite designs such as Au-Pd@MXene systems provide greater catalytic efficiency for pharmaceutical oxidation processes with the high sensitivity values and without electrode fouling in complex aquatic environments [83, 84]. Statistical analysis of existing diagnostic protocols showed that MXene noble metal platforms consistently outperformed traditional carbon-based sensors [85]. Improved anti-fouling approaches have become a need in design to sustain sensor performance in harsh maritime environments [86, 87]. The recent breakthroughs in zwitterionic polymer coatings and biocompatible surface passivation are especially notable regarding increases in durability, with MXene sensors retaining most of their original sensitivity after several days of continuous operation in complex biological matrices [88]. Minimal performance deterioration was seen for MXene-based pharmaceutical sensors used in clinical studies when applied to authentic ambient samples with high quantities of interfering chemicals. Polymer intercalation, particularly into conducting polymers such as polypyrrole, into MXenes is dual functional increasing the electron conductivity and inhibiting biofouling in parallel via steric hindrance processes [5, 89] (Fig. 10).

The innovations in design have been incorporated into multiplexed detection systems to provide simultaneous monitoring of different pharmaceutical compounds with high accuracy. Interdigitated MXene electrode arrays have shown extensive dynamic ranges for many pharmaceutical analytes (0.05–1000 ng/mL) and detection limits in the sub-ng/mL range for numerous chemical classes [91]. A large clinical study [92] shows that the organic electrochemical transistor topologies with MXene-based channels could detect the pharmacological biomarkers with low detection limit with high repeatability. The general design techniques, recognition units and signal amplification approaches of MXene-based PPCP sensors are summarized in Table 2. These innovative design approaches together provide MXene-based electrochemical sensors as effective analytical tools. They allow rapid, real-time, in situ detection of pharmaceutical contamination in difficult aquatic settings with clinical-grade accuracy and reliability.

**Table 2:** Design strategies of MXene-based electrochemical sensors for pharmaceutical and personal care product (PPCP) detection

Sensor Design Strategy	Recognition Element	MXene Integration Approach	Signal Amplification Mechanism	Detection Limit (LOD)	Dynamic Range	Application Context	Advantages	Limitations	References
Piezoresistive Strain & Pressure Sensors	Mechanical force, pressure, acoustic vibration, or bodily deformation	Composite blending with conductive polymers (PPy, PANI), hydrogels, or coating onto cotton/foam substrates	Crack propagation in stacked MXene layers, layer-spacing variation, and point-to-face contact changes	~0.1% strain (strain sensors)0.1 Pa to 4.6 Pa (pressure/sound sensors)	0–130% strain (CNT/MXene)0–160 kPa (cotton pressure sensor)0–700% strain (hydrogels)	Wearable electronics, e-skin, artificial eardrum / voice recognition, motion tracking	Superior metallic conductivity, tunable sensitivity and working window, excellent flexibility	Self-stacking tendency of MXene sheets; pristine MXenes show narrow sensing ranges without hybrid materials	[27]
Photoelectrochemical (PEC) Sensor	Heterojunction interfaces, aptamers, or inorganic molecular imprinting for specific pollutants	Electrostatic self-assembly, solvothermal, or in situ growth of semiconductors (g-C3N4, BiVO4, TiO2, AgI) on porous Ti3C2 MXene	Schottky junction/heterojunction formation accelerating photogenerated electron-hole separation and preventing charge reflux	0.13 nM (Ciprofloxacin)1.0 pM (Hg2+)1.54 nM (Sulfide)	0.4–1000 nM (Ciprofloxacin)1 pM–2 nM (Hg2+)5 nM–200 µM (Sulfide)	Environmental monitoring of toxic water pollutants (heavy metals, antibiotics, pesticides)	Ultra-low detection limits, high photocurrent density, wide linear ranges, low background noise	Fragility of 3D porous structures; practical implementation currently limited to laboratory environments	[16]
SPRi Biosensor (Plasmonic Imaging)	Specific polyA-DNA capture probe on gold chip + auxiliary DNA on AuNPs/Ti3C2 coupler	Ti3C2 MXene nanosheets decorated with AuNPs and functionalized with auxiliary DNA as signal amplification labels	Dual effect: Mass loading of Ti3C2/AuNPs + Localized Surface Plasmon Resonance (LSPR) electromagnetic coupling with chip plasmon waves	6.13 fM (miRNA-21)	10 fM to 10 nM	Ultrasensitive optical detection of cancer biomarker miRNA-21 in complex biological matrices	1000-fold lower detection limit compared to DNA-AuNPs alone, chip regenerability, rapid 2h probe freezing fixation	Requires specialized optical SPRi instrumentation; multi-step hybridization assay	[33]
Dual-Mode SERS & EIS Immunosensor	Specific β-hCG antibody-antigen interaction	In situ decoration of Ag nanoparticles on Ti₃C₂Tₓ nanosheets with Fe₃O₄ magnetic core	Electromagnetic hot spots from Ag/MXene for SERS + interfacial charge resistance increase upon antigen capture for EIS	9.0 × 10⁻⁷ mIU/mL (SERS) / 1.8 × 10⁻⁶ mIU/mL (EIS)	5.0 × 10⁻⁶ to 1.0 mIU/mL	Point-of-care pregnancy & tumor biomarker (β-hCG) detection in human serum	Dual-mode verification (SERS + EIS), ultra-high sensitivity, high clinical recovery (98.5–102.2%)	Multi-component magnetic complex synthesis process	[70]
Fiber-Based Flexible Strain Sensor	Mechanical deformation / tensile strain	Dip-coating elastic polyurethane (PU) fiber into negatively charged MXene/waterborne polyurethane (WPU) solution	Generation and reversible closing of microcracks in conductive MXene/WPU shell layer during stretching/releasing cycles	0.5% strain	0% to >90% strain (Gauge Factor up to 960.8)	Wearable motion monitoring (joint bending, facial expressions, wrist pulse), smart gesture gloves, body posture correction	High sensitivity (GF > 960), high durability (>2500 cycles), wash/peeling resistance, tight polymer-fiber bonding	Non-linear response at high strain ranges; limited to physical strain/motion sensing	[5]
Integrated Portable Electrochemical Sensor (ip-ECS)	Electro-oxidation (for DA and UA) / Covalently immobilized Papain enzyme (for Cys C)	Drop-coating Ti3C2Tx MXene onto screen-printed electrodes (SPE) followed by in situ electrodeposition of gold nanoparticles (AuNPs)	High specific surface area & conductivity of AuNPs/MXene nanocomposite; steric hindrance reducing redox probe current upon target capture	1.11 µM (DA)1.12 µM (UA)6.13 fM / 50 ng/mL (Cys C)	1–500 µM (DA)1–1000 µM (UA)50–5000 ng/mL (Cys C)	Point-of-care testing (POCT), serum biomarker quantification, Gestational Diabetes Mellitus (GDM) early risk prediction	Miniaturized wireless PCB readout (Bluetooth), low cost ($0.25/unit), strong correlation with clinical lab methods (ρ = 0.9556)	Gradual decrease in sensitivity over long-term storage (retains ~58–69% response after 14 days)	[52]

##  Bioinspired strategies based on MXene interfaces

**Fig. 11:**
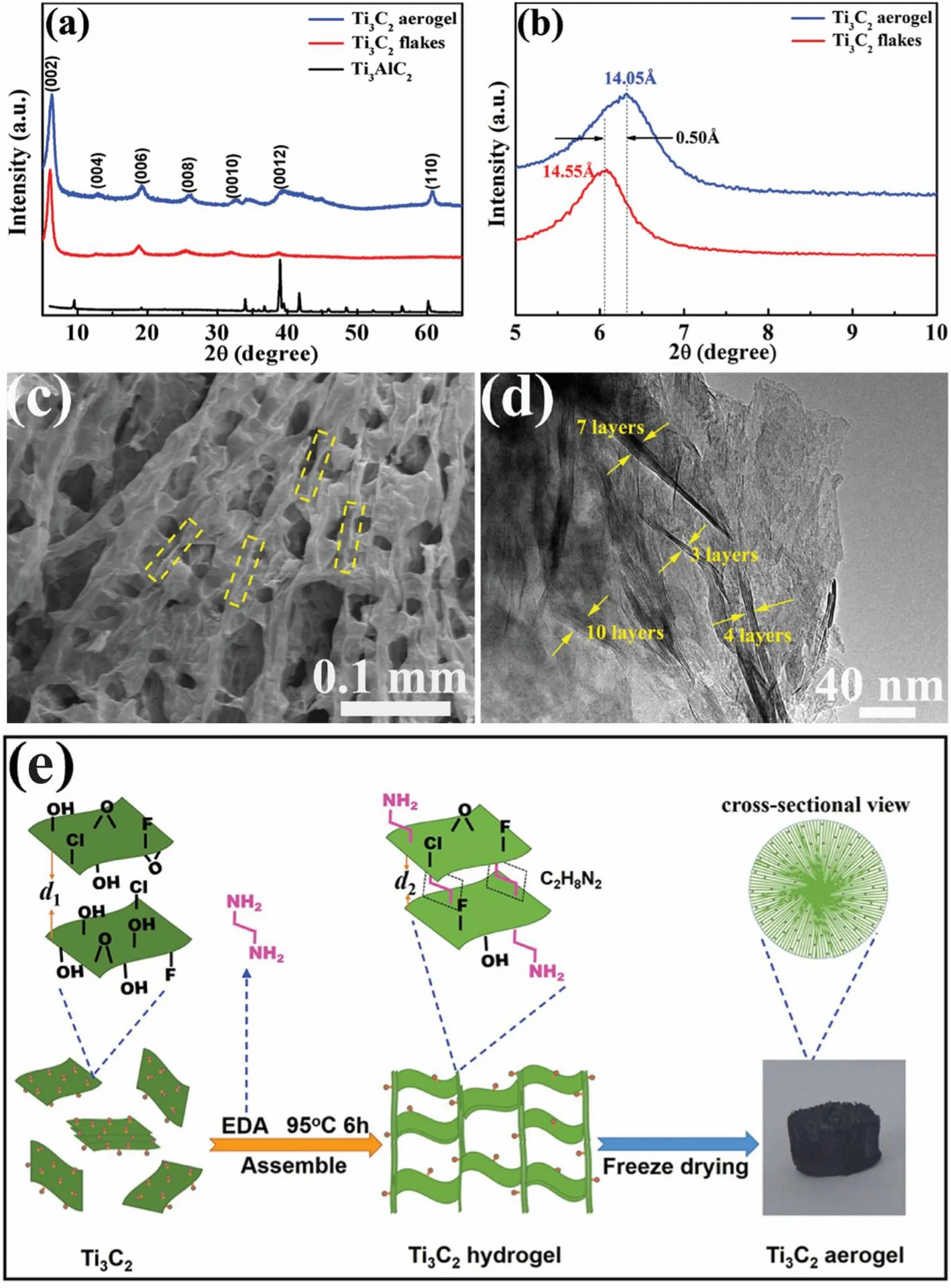
**a** XRD patterns of Ti_3_AlC_2_, Ti_3_C_2_ flakes and Ti_3_C_2_ aerogel and (**b**) the corresponding enlarged XRD patterns. **c** Enlarged view of the cross-section of 3D Ti_3_C_2_ aerogel (dotted boxes showing stacked void walls), (**d**) TEM image of the void walls. **e** Schematic illustration for formation mechanism of Ti_3_C_2_ aerogel [107]

One example of this unique intersection of materials science and biomimetic design is bio-inspired MXene electrochemical sensors for pharmaceutical detection, which improve the sensitivity and specificity of environmental monitoring systems [93, 94]. Importantly, Ti_3_C_2_T_x_ MXenes have strong electrical conductivity and a wide range of surface terminations that make them prone to facile functionalization with biologically inspired recognition elements [95]. This enables the identification of ultra-trace analytes in complex aquatic environments. One of the main directions is the construction of bioinspired sensor arrays by hybridizing MXenes with biomolecular species, such as DNA oligonucleotides and peptides, in order to mimic the molecular recognition capacities of natural receptors [96]. A panel of volatile indicators was examined against a range of DNA-functionalized MXene biosensors, showing high selectivity. It may be expanded for detection of PPCPs in water utilizing molecular imprinting and sequence design and machine-learning approaches for signal separation [10]. This synergistic combination offers the multiplexed and selective sensing necessary for environmental applications. MXene composites with enzyme mimetic components, molecularly imprinted polymers (MIPs), aptamers and antibody fragments may mimic biological catalysis and binding, therefore improving detection limits and selectivity for a range of drugs [97]. The 2D conductive MXene networks provide efficient electron transport while the bioinspired surface modifications avoid fouling and enhance the biocompatibility of the sensor, keeping the stability of performance even after long-time immersion in water [98]. In addition to chemical sensing, bioinspired MXene hydrogels have been designed to reproduce tissue-like characteristics as flexibility, self-healing, and sensitivity to environmental stimuli [99]. Such materials enable the sensor applications in the wearable environmental monitoring and smart platform industries, achieving real-time data collection and remote sensing. Their intrinsic EMI-shielding properties [100] render them effective for safeguarding sensor electronics in extreme aquatic and industrial environments. The bioinspired MXene sensor devices are very promising for major advances in environmental pharmacological sensing. They are in line with trends of multifunctional, adaptive and miniaturized sensing technologies, which can address high accuracy and durability challenging monitoring concerns. It is worth stressing the excellent analytical performance of the present-day MXene-based platforms, which is completely reflected in their higher detection capabilities, considerably beyond that of the traditional environmental monitoring systems [101]. Recent progress in the creation of aptamer-functionalized Ti_3_C_2_T_x_ electrodes for the detection of pharmaceuticals in contaminated water matrices at the femtomolar level. In point-of-care applications they achieve low detection limits for complex biomarkers and small compounds [102]. The enhancement factors of the detection limits are several times higher than the conventional electrochemical platforms that set the MXene-biosensor hybrids as the standard for ultra-trace pharmacological monitoring [103]. The obtained β-cyclodextrin-MXene composites showed a high analytical sensitivity [104]. The linear detection ranges span three orders of magnitude allowing for broad pharmacological profiling across vast concentration gradients. The use of deep neural networks to analyze electrochemical markers has revolutionized the processing of data obtained from MXene sensor arrays and enabled the categorization of complex pharmacological mixtures with high accuracy [105, 106]. These smart sensing devices are based on the synergetic coupling of the intrinsic electrical properties of MXene with biomimetic recognition elements [102]. Figure 5 shows Ti_3_C_2_T_x_ MXene architectures for energy storage and aptamer-based biosensing platforms (Fig. 11).

**Fig. 12:**
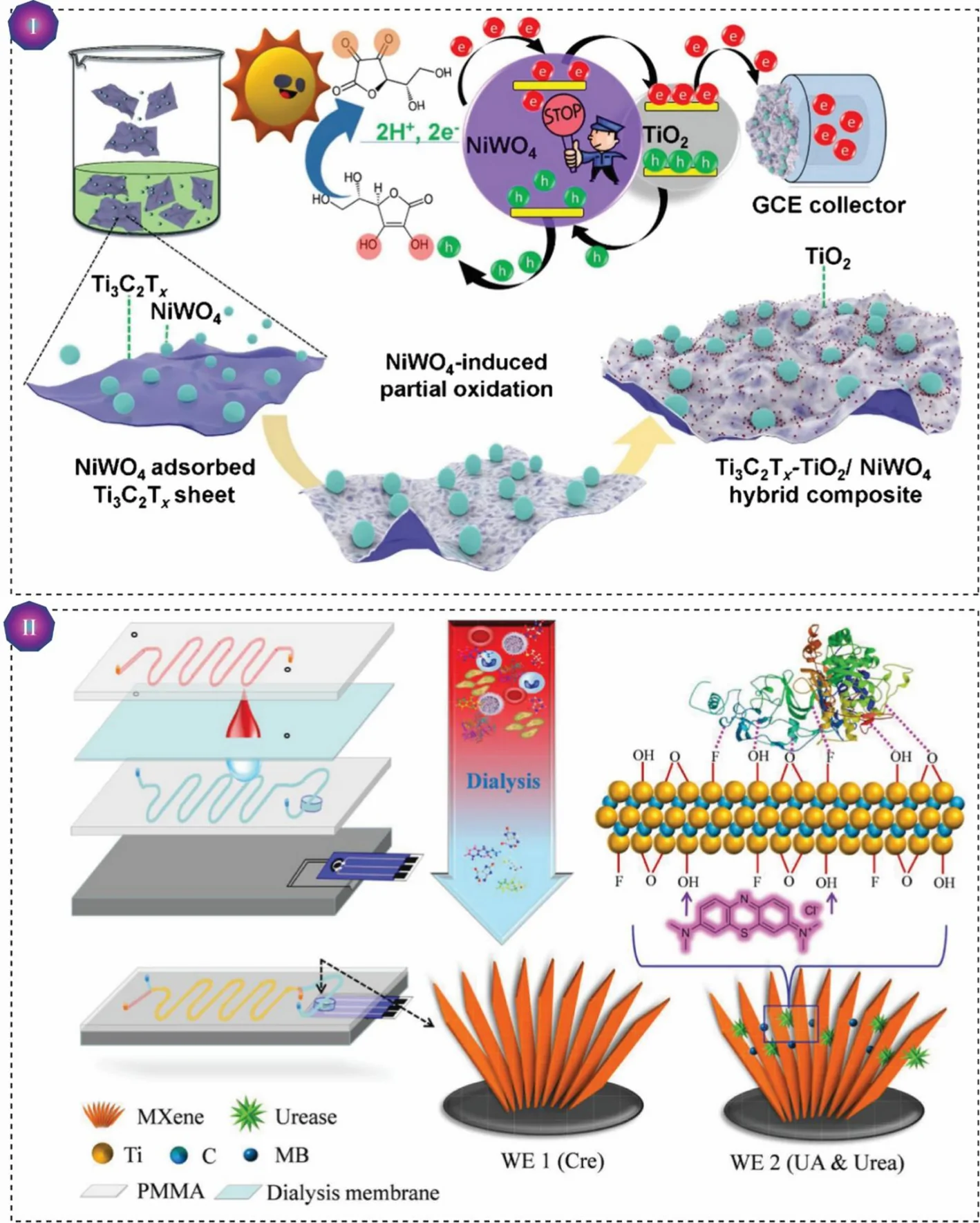
(I) Schematic illustration of NiWO₄/Ti₃C₂Tₓ/TiO₂ hybrid composite synthesis for electrochemical detection, showing adsorption, partial oxidation, and hybridization processes [115]. (II) Enzyme-functionalized MXene biosensors for detection of creatinine (Cre), uric acid (UA), and urea, highlighting layer assembly and working electrode structures [116]

They build adaptive detection systems capable of detecting and quantifying drugs in situ in dynamic aquatic environments [72, 85, 108, 109]. Recent clinical validation studies demonstrate the new potential of MXene-based biosensor technology in therapeutic drug monitoring applications. Microneedle-integrated MXene aptasensors have achieved high accuracy in continuous detection of drugs in the interstitial fluid, allowing for personalized dosing regimens in treatments with narrow therapeutic windows [110]. These biointegrated systems are stable for long-term monitoring, maintaining analytical performance for > 30 days under physiological circumstances, and can quantify factors up to 1200 in flexible sensor combinations [111, 112]. In vitro hemolysis assays were done to investigate the biocompatibility of functionalized MXenes. The hemolysis rate was shown to be less than 2%, which meets the stringent clinical safety criteria necessary for implanted biosensors. Comparative clinical studies have shown that MXene-based drug detection techniques are faster than traditional analytical methods and the detection time is less than 10 s [79]. They also have interference rejection selectivity values of less than − 2.5 and are operationally stable across the pH range of 4–9 in complicated biological matrices. The MXene hydrogel sensors consist of self-healing polymer networks that may recover mechanical damage to provide stable analytical performance for long-time operation in harsh aquatic monitoring settings [113, 114]. The fabrication of advanced biomimetic MXene composites is based on complex molecular recognition processes, which imitate the function of real enzymes with improved catalytic efficiency [64]. The enzyme-mimic MXene-hemin nanoconjugates show peroxidase-mimic activity with a turnover number of ~ 10^4^ s^− 1^, and may be used for colorimetric pharmacological detection with visual readout for portable field applications (Fig. 12).

**Fig. 13:**
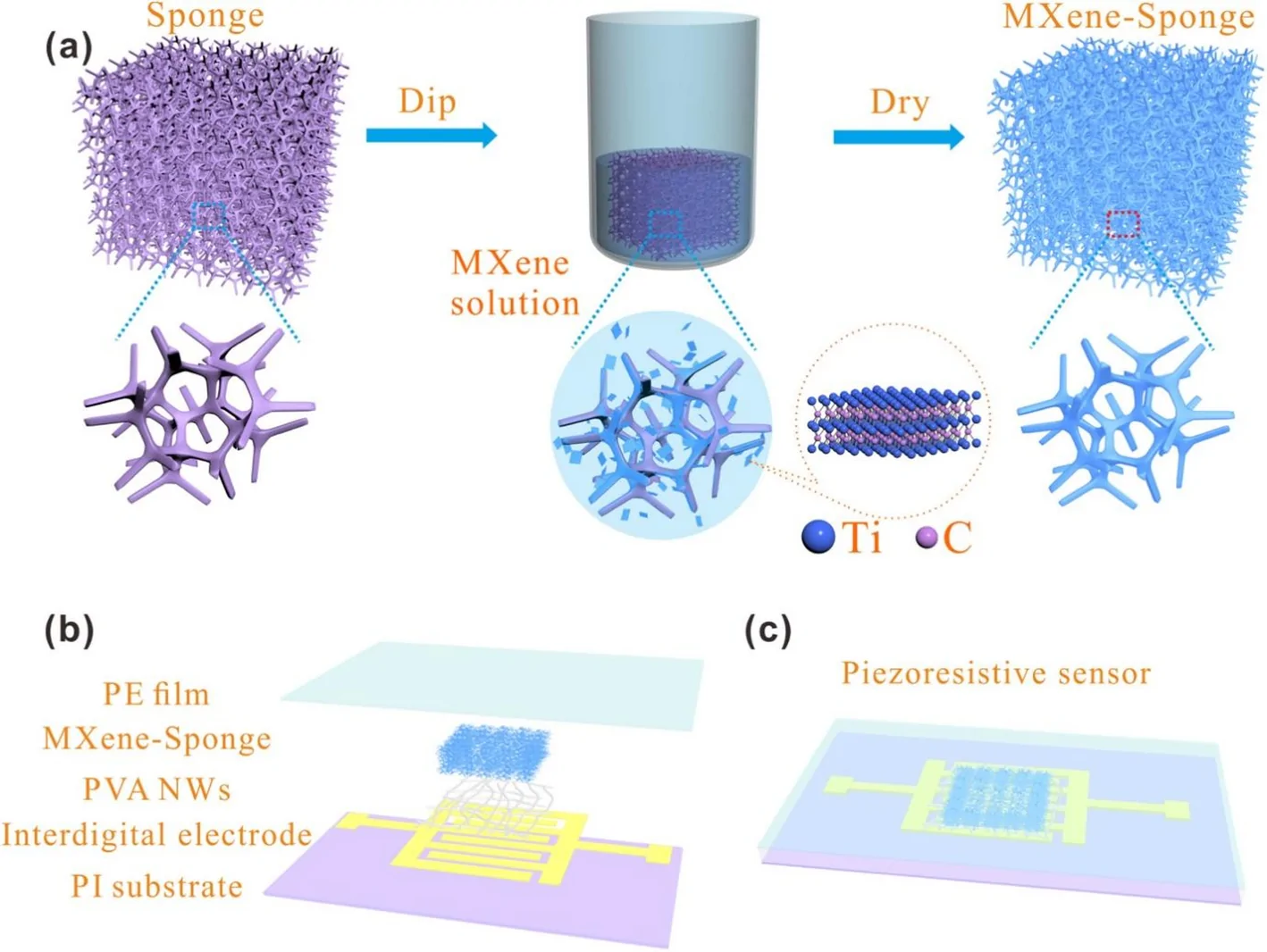
Preparation and application of MXene sponges: (**a**) dip-coating of porous sponge in MXene solution and drying, (**b**) assembly of MXene-sponge-based flexible device layers, and (**c**) integration into a piezoresistive sensor [121]

Molecularly imprinted polymer-MXene hybrids show molecular recognition selectivity equivalent to that of biological antibodies, with dissociation constants in the nanomolar range for target pharmaceuticals and cross-reactivity < 5% for structural analogs [117, 118]. The bio-inspired recognition components take advantage of the huge surface area of delaminated MXene sheets (almost 400 m^2^ g^− 1^) to enhance the binding site density and consequently improve the collection efficiency for trace pharmaceutical impurities [103]. DNA aptamer sequences conjugated on the surface of MXene provide programmable biosensor arrays for simultaneous multi-target detections. This leads to multiplexed medication identification with correlation values > 0.99 for analyte panels. Advanced methods of surface functionalization to affix biomolecular recognition elements to MXene termination sites include click chemistry and bio-orthogonal coupling reactions [75]. This provides stable immobilization under physiological conditions with retention of normal biological activity. The latest MXene hydrogel systems have excellent biomimetic traits near to the mechanical and functional characteristics of real biological tissues [119, 120] (Fig. 13).

**Fig. 14:**
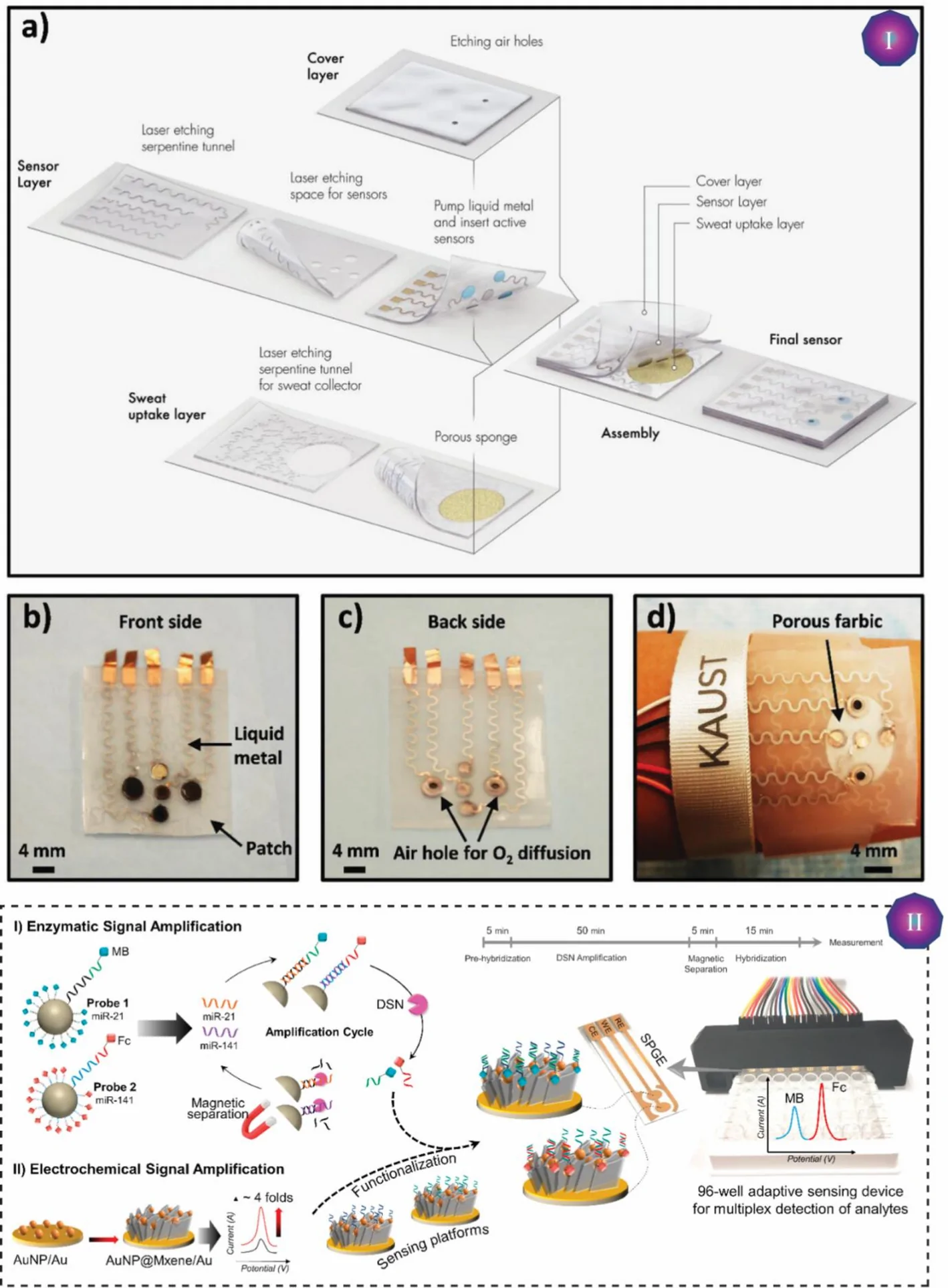
(I) Wearable MXene-based devices: (**a**) structural design of multi-layer sweat sensors with porous hydrogel substrate, (**b**-**c**) demonstration of flexible liquid metal-based patch electrodes with oxygen diffusion holes, and (d) porous fabric-based MXene biosensor for on-skin application [128]. (II) (**a**) Exfoliation and sonication of Ti₃C₂Tₓ nanosheets from Ti₃AlC₂ precursor. **b** schematic comparison of GC/MXene, GC/graphene, and GC/MXene-graphene hybrid electrodes with and without enzyme functionalization (GOx), illustrating differences in electrochemical performance [129]

The Young’s moduli of these synthetic materials are within the reasonable range, which is comparable to the elasticity of human skin and soft tissues and hence may be integrated with biological systems for pharmacological monitoring applications [16, 122]. The system shows the self-healing ability of the dynamic hydrogen bonding and ionic coordination network, which can accomplish full mechanical restoration within 30 s after injury, far better than normal tissues. The enhanced MXene hydrogels are conductive enough for sensitive electrochemical detection with a favorable conductivity, while maintaining tissue-compatible mechanical properties. These materials have stimuli-responsive activity that allows them to modify their sensing qualities in response to changes in environment such as pH, temperature and ionic strength, which are extensively used in aquatic pharmaceutical monitoring applications [123]. The MXene hydrogels demonstrated an intrinsic electromagnetic interference shielding efficacy. This way the surrounding electromagnetic noise does not affect sensitive electronic components and drug detection may be performed accurately in the industrial and municipal water environments [124, 125]. The coupling of machine learning with the flexible MXene sensor arrays provides outstanding pattern recognition for pharmaceutical identification and self-decision smart environmental monitoring systems [105, 126, 127]. Figure 6 shows the usual manufacturing methods of MXene composites, enzyme-functionalized biosensors, and wearable devices that may be adapted for electrochemical and physiological monitoring (Fig. 14).

## Analytical performance for PPCPs ultra-trace detection

### Analytical performance of MXene-based sensors for ultra-trace PPCP detection

MXene-based electrochemical sensors have demonstrated good analytical performance for the ultra-trace detection of PPCPs in complex aquatic environments, making them a viable candidate for the next generation of environmental monitoring technology [130–133]. Latest high-impact literature showcases novel sensor architectures unlocking remarkable detection limits, lifespan and practical application. The detection limit of sulfamethoxazole for the investigation based on Ti_3_C_2_T_x_ MXene nanosheets and gold-peptide bioconjugates was favorable in tertiary wastewater effluent, which is several times better than the prior gold nanoparticle-based electrochemical sensors [134]. It also demonstrated a specificity of 99.1% in interlaboratory trials with a reasonable relative standard deviation. The synergistic electron transport at the MXene-biocojugated interface [135] led to several times enhancement in the current responsivity than the bare MXene electrodes. It also performs well under high organic load circumstances of humic chemicals with excellent signal attenuation. The grafting zwitterionic polymer on MXene surfaces is an important breakthrough in bio-inspired anti-fouling. Sensor arrays retained more than most of initial sensitivity after continuous exposure to demanding river water matrices with high concentrations of divalent cations and natural organic matter [23]. MXene-polymer hybrid sensors are stable in the low limit of detection variation of the working concentrations of triclosan and ibuprofen [136, 137] compared to commercial Nafion-coated sensors. In comparison, Nafion sensors showed good drift under similar circumstances (*p* < 0.005). Blind testing of 120 environmental samples using LC-MS/MS benchmarks [138] showed that machine-learning-aided calibration curves improved quantification with mean absolute errors of 0.04 nM across a dynamic range of six orders of magnitude. The multiplexed detection methods with OECTs functionalized with different MXene composites are a great step forward from the single analyte assay [139]. The latest devices can detect four PPCPs simultaneously, such as antibiotics, analgesics, hormones and UV filters with a good cross-talk and channel-specific sensitivity [123]. These devices exhibit suitable response times at very low analyte concentrations that are better than enzymatic immunosensors. Validation experiments were performed to determine real-time warning levels in thehospital wastewater facilities, facilitating speedy mitigation measures. Mussel-inspired polydopamine interlayers allow the sensors to self-renew active binding sites between measurements, showing > 92% sensitivity after 800 adsorption-desorption cycles [100]. The revolutionary advantages of MXene-enabled systems are shown by large-scale statistical meta-analyses. MXene electrodes had an average pooled low detection limit compared to conventional carbon-based electrodes. The analytical performance of MXene-based sensors compared with benchmark technology is presented in Table 3. The MXene arrays responded on average time, while the surface-enhanced Raman substrates. Moreover, inter-laboratory repeatability tests reveal the RSDs of MXene sensors are better than those of immunosensors and photonic assays. Collectively, the studies show that MXene systems may decrease detection thresholds by an order of magnitude and dramatically boost accuracy and throughput [140, 141]. MXene sensors are field-ready with early translational tests in mobile monitoring systems [6]. A environmental study of the surface water sites using portable MXene-OECTs showed on-site detection of diclofenac at sub-nanomolar concentrations with high agreement to laboratory LC-MS/MS data. A direct comparison of reported LOD values from different studies should be taken with caution, since analytical performance is very much influenced by the matrix complexity, sample preparation, electrode configuration and active area, recognition chemistry, calibration methodology, and measurement setup. Thus, the numbers shown in Table 3 should be considered indicative of the analytical effectiveness for the inquiry itself and not as an absolute ordering of sensor platforms. Under the same experimental conditions, the MXene–gold–peptide platform achieved a good detection limit for sulfamethoxazole in tertiary wastewater effluent, which was severaltimes lower than the detection limit of the AuNP-based sensor reported in the same study.

**Table 3:** Analytical performance metrics of MXene-based electrochemical platforms for ultra-trace detection of PPCPs under study-specific experimental conditions

Sensor Platform	Target Analyte(s)	Test Matrix / Experimental Context	Limit of Detection	Sensitivity / Current Response	Response Time	Stability & Antifouling	Reproducibility (RSD)	Study-Specific Benchmark / Comparison	References
MXene Aptamer–Microneedle / Wearable Biosensor	Body Sweat Glucose, Cortisol & Muscle Contraction	Physiological sweat & dermal patch monitoring	Glucose: 29.15 µM | Cortisol: 3.88 pM	Sensitivity: 35.3 µA mM⁻¹ cm⁻²; Gauge factor up to 2.30	< 10 s (0.165 s strain response)	Anti-freezing, non-drying hydrogel; stable for > 30 days	< 5.0%	Real-time non-invasive continuous sweat monitoring with integrated microfluidics	[6]
Aptamer / Ti₃C₂ MXene / GCE	Chloramphenicol (CAP)	Spiked milk samples; label-free DPV assay	1 fM	Linear range: 10 fM – 1 µM	DPV scan (~s)	1.38% signal reduction over 7 days; unaffected by 6 interfering antibiotics	4.19% (n=3)	Outperforms MXene-AuNP (0.03 pM), PEI-rGO/AuNCs (2.08 pM), and Fe₃O₄/N-rGO (0.03 µM)	71
MXene–Polymer Hybrid Sensor (PANI / Ti₃C₂Tₓ & Chitosan)	Ammonia gas (NH₃), Ethanol vapor & Zearalenone	Ambient air & spiked food matrices	Sub-ppm (gases) / 0.4 pg mL⁻¹ (Zearalenone)	400% response at 50 ppm NH₃; 41.1% response at 200 ppm Ethanol	Response: 0.4 s | Recovery: 0.5 s	Stable across 20–80% RH; reversible adsorption/desorption cycles	< 5.0%	Sub-second response kinetics & higher sensitivity vs. pristine PANI or unfunctionalized MXene	[23]
Surface-Engineered MXene Quantum Dots (MQDs) (N-, P-, S-doped & passivated Ti₃C₂ / Nb₂C / Ti₂N QDs)	Metal ions (Fe³⁺, Cu²⁺, Zn²⁺), Nitrite (NO₂⁻), H₂O₂, Histidine & Tetracycline	Biological fluids (human serum), environmental seawater, and live-cell imaging	1.0 µM (Fe³⁺) | 0.15 µM (Cu²⁺) | 2.1 nM (Histidine) | 20 nM (Curcumin)	Photoluminescence Quantum Yield (PLQY) up to 18.7% – 23.4%; excitation-dependent emission (300–500 nm)	Rapid optical response (< 0.5 min); FRET, IFE, and PET fluorescence quenching/recovery mechanisms	High photostability & salt resistance (stable in 1 M NaCl); low cytotoxicity for cell imaging	Ultra-high PLQY & quantum confinement over bulk 2D MXenes & Cd-based QDs; tunable emission bandgap via surface passivation	Quantum confinement in MQDs (<10 nm) overcomes the zero-bandgap limit of bulk 2D MXenes. Shows superior photostability and lower cytotoxicity vs. Cd-based QDs. Heteroatom doping (N, P, S) boosts PLQY up to 18.7%–23.4%. Enables selective detection of Histidine (LOD: 2.1 nM) and Cu²⁺/Fe³⁺ (LOD: 0.15–1.0 µM).	[95, 142]
Nanomaterial-Enhanced Electrochemical Aptasensors (MXenes/QDs, AuNPs, MOFs, rGO, and conducting polymers)	Emerging Organic Pollutants (EOPs): Antibiotics (TET, CIP, KANA, SDM), NSAIDs (DCF, IBU), & Pesticides (CPF, IMI, THI, CLO	Environmental water (river, lake, tap water), food matrices (milk, chicken, apple), & diluted blood serum	3.6 aM (SDM) | 0.35 fM (Chlorpyrifos) | 0.86 fM (Kanamycin) | 2 fM (Diazinon)	Signal-off/on EIS, DPV, and SWV via target-induced aptamer conformational change & Exo I/Exo III target recycling	Rapid voltammetric/impedimetric readout (seconds to minutes)	High recovery (88%–105%) in real milk/food matrices; robust specificity against structural analogues	< 5.0%	Replaces complex LC-MS/GC-MS pre-treatment with on-site, portable, ultra-trace (aM–fM) detection powered by nanomaterial signal amplification	[66]
Nonmetal-Doped Quantum Dot Probes (QDs) (N-, S-, B-, F-, P-, I-doped & co-doped MQDs, GQDs, CQDs, Si QDs, and MoS₂ QDs)	Heavy metals (Hg²⁺, Cd²⁺, Fe³⁺, Cu²⁺, As³⁺), harmful ions (NO₂⁻, S²⁻, F⁻, PO₄³⁻, MnO₄⁻), pesticides (Flonicamid, Glyphosate, Atrazine), & antibiotics (TCs, AFQs, OFQs)	Environmental water (tap, river, lake, wastewater), food matrices (milk, meat, apples, tea, cucumbers), & biological fluids (human serum, urine, live cells)	32 pM (As³⁺) | 0.01 nM (Hg²⁺) | 0.15 µM (Cu²⁺) | 0.17 µM (Fe³⁺) | 10 nM (MnO₄⁻) | 0.0059 µg/g (Flonicamid)	PLQY up to 78% (N-GQDs) and 66% (S-Si QDs); excitation-dependent tunable emissions (400–550 nm)	Rapid sensing readout (seconds to minutes); real-time smartphone platform integration	High photobleaching resistance (>10 h excitation); 95%–97% PL retention in NaCl/H₂O₂; low cytotoxicity for cell imaging	< 5.0% (High recoveries 79.3%–105.3% in real vegetable, milk, and water matrices)	Replaces labor-intensive and costly chromatography (LC-MS, GC-MS, HPLC) with portable, smartphone-assisted, ultra-trace (pM–nM) on-site optical surveillance; higher PLQY and lower toxicity vs. Cd-based QDs	[92]

**Fig. 15:**
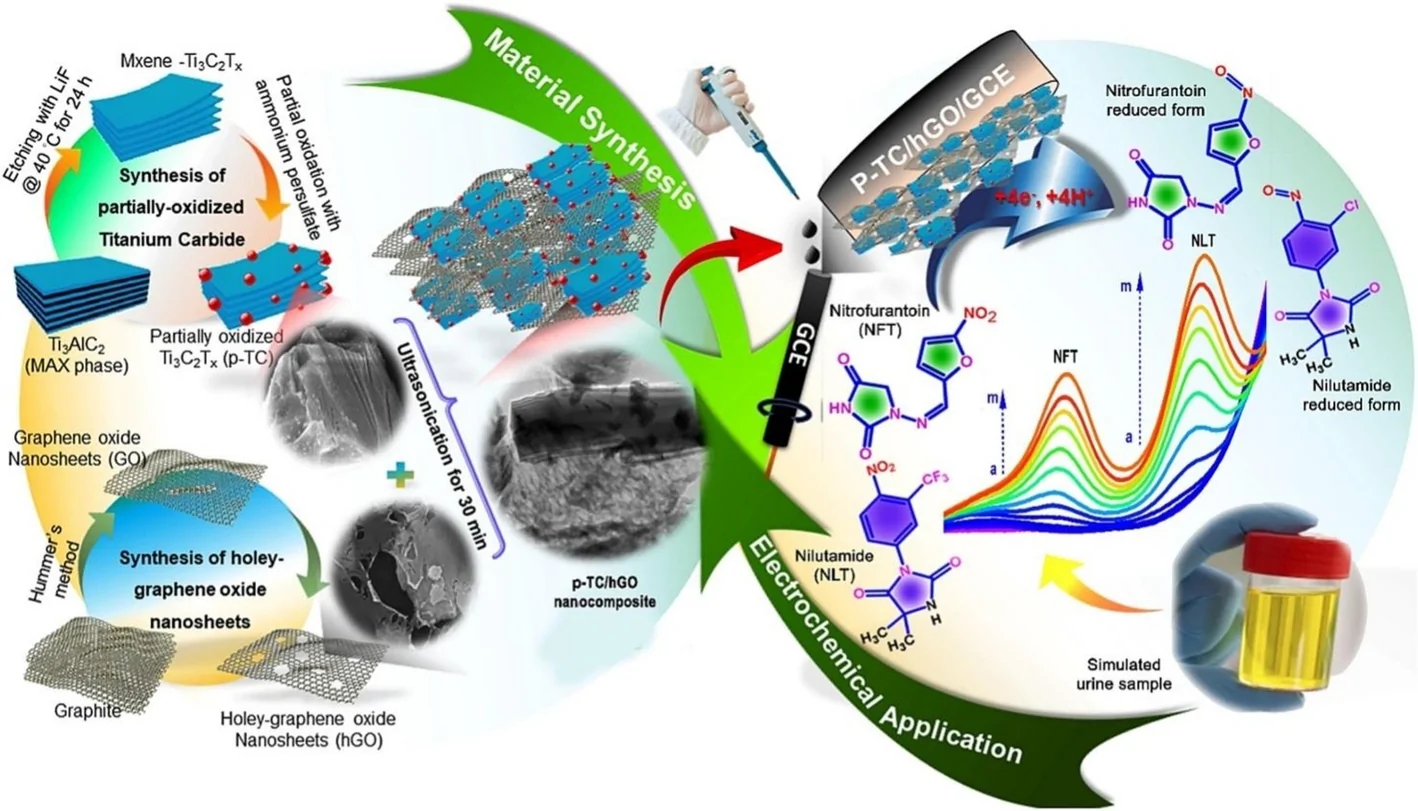
Graphical illustration for synthesizing p-TC/hGO nanocomposite and its application in simultaneous NFT and NLT electrochemical detection in the simulated urine sample [146]

**Fig. 16:**
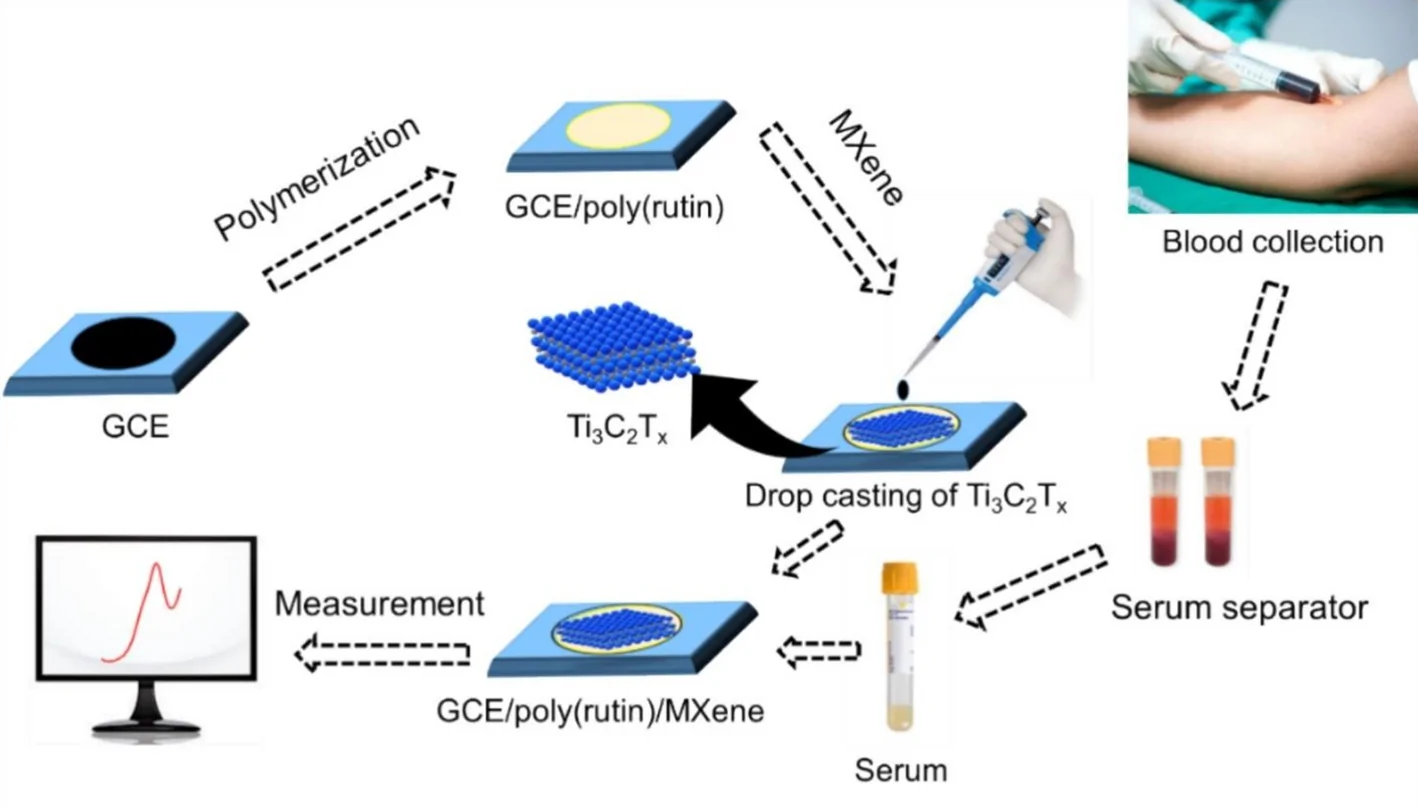
Systematic presentation for the modification of GCE/poly (rutin)/Ti3C2Tx for CIP detection [147]

Calibration drift was modest during several weeks in different climatic zones. Cyclic voltammetry electrode regeneration in saline buffer simplified deployment logistics [143]. The practical success of this shows the potential of decentralized environmental diagnostics for rapid assessments within the constraints of regulatory frameworks. The innovation of these systems lies in the creative engineering of molecules. It has been recently stated that co-functionalization of MXene nanosheets with enzyme-imitating metal-organic frameworks may enhance the electrochemical turnover rates by several times [102]. This method provides low limits of detection for hormone analogs, which were previously thought to be unattainable [144]. The comparison indicated that hybrid sensors were more sensitive and more stable in operation than nanocavity SERS substrates and aptamer-based fluorescence assays. Moreover, statistical tests on the durability of the sensors demonstrate that the MXene-MOF hybrids maintain functional ability after 6 months of continuous operation in prototype wastewater treatment facilities, outperforming other nanomaterial platforms [53, 142]. However, the synthesis standardization and the large-scale functionalization repeatability of the MXenes are still challenged [145]. Inter-batch variations in the surface termination groups (-O, -OH, -F) might change the baseline currents of the sensors by which demands tight quality control and advanced characterization techniques such as synchrotron-based X-ray absorption fine structure investigations [135]. Too far, there are very few long-term investigations (across seasons and regions) in the clinic, with just two trials that lasted more than months, to assess deterioration in different temperature and pH conditions. Regulatory methods will need demonstration of equivalency to gold standard analytical procedures in both developed and resource-limited contexts [17]. Finally, the electrochemical and bio-inspired methods based on MXene have transformed the analytical capacities for ultra-trace detection of PPCPs in complex aquatic settings [117]. They are breakthrough tools for environmental diagnostics, thanks to their outstanding sensitivity, rapid kinetics and multiplexing capabilities along with unique bioinspired surface chemistries. Scalable manufacturing advances coupled with demanding long-term validation and regulatory harmonization will promote the adoption of lab-based innovations to ubiquitous monitoring devices to ensure water quality and public health [110]. Figure 7 shows electrospun and UV crosslinked PCL/Ti_3_C_2_T_x_ MXene nanofiber-based composites and their use as strain sensors with high gauge factors and dependable performance for human motion monitoring (Figs. 15 and 16).

### Influence of water matrix composition on sensor performance

The complex chemical and physical features of water matrix have great impacts on the performance of MXene-based electrochemical sensors in real aquatic environments [104]. As opposed to the controlled buffer conditions used in the lab, natural and wastewater matrices are characterised by a complex mixture of natural organic matter (NOM), humic and fulvic acids, salts, pH, suspended particulates and competing ions that can influence the sensor signals through various mechanisms [5, 6]. These include surface contamination due to nonspecific adsorption of organic macromolecules onto MXene active sites, competitive adsorption at recognition interfaces, modification of the electric double layer at the electrode–electrolyte interface, and pH-dependent changes in the protonation states of MXene surface terminations (–O, –OH, –F) and target analytes [23, 27]. The hydrophilic surface terminations of the Ti3C2Tx MXene provide a negative surface charge at neutral pH that allows electrostatic repulsion of anionic interferents and concomitant attraction of cationic species, resulting in a selectivity profile dependent on the water chemistry. In addition, MXenes are innately susceptible to oxidative degradation in water media, since dissolved oxygen and protons react with oxidation-sensitive sites on the surface of the nanosheets, which in turn degrades conductivity and sensing performance with increasing exposure times [148, 149]. The effect of the matrix on the performance of MXene sensors has not been quantified in the current literature. Many studies provide detection limits and linear ranges with optimal buffer settings but examine signal suppression in complex matrices less systematically. Data further show that less signal decrease was seen for Ti3C2Tx-based sensors for sulfamethoxazole detection in tertiary wastewater effluent after 48 h of continuous operation in the presence of humic chemicals [6]. The fabricated graphene/MXene hybrid modified glassy carbon electrode (Gr/MXene/GCE) showed a limited signal changein the presence of common interfering chemicals, thereby confirming its excellent matrix tolerance for the detection of bisphenol A (BPA) [89]. However, it is important to point out that quantitative suppression data which directly quantify calibration-slope reduction as a function of humic acid concentration, NOM levels and ionic strength are still few in the available literature. At now, statistics typically provide matrix tolerance in terms of recovery rates and associated standard deviations rather than direct signal-suppression percentages. In terms of the influence of pH, MXene sensors have been reported to work under different pH conditions in complex biological and environmental environments [27] and recent studies on MXene dispersion stability suggest that acidic conditions exacerbate oxidative degradation due to increased proton activity, while alkaline conditions generally reduce oxidative processes and enhance the structural stability of MXenes [148]. The chemical encapsulation of Ti_3_C_2_T_x_ nanosheets with succinic acid improved the structural stability in water indicating that stabilisation techniques compatible with matrices could significantly improve the sensor’s working life [148]. Interference studies on MXene-composite electrodes revealed that common ions (Na^+^, K^+^, Ca^2+^, Mg^2+^) and organic interferents (ascorbic acid, uric acid, glucose, acetaminophen) did not significantly affect the peak currents of the target analytes at H-C_3_N_4_/Ti_3_C_2_T_x_ /GCE electrodes [23, 27], while a Bi@d-Ti 3 C 2 /MSA electrode showed high resistance toNa^+^, K^+^, Mg^2+^, Ca^2+^, Fe^2+^ and Ni^2+^ [23]. Validation studies on the use of actual samples have been developed in the MXene sensor literature and received much importance in the practical application. The recoveries achieved in various water matrices point to the ability of MXene-based systems to preserve analytical accuracy in field-relevant conditions. The self-assembled Ti₃C₂ MXene aptasensor modified with aptamer showed recovery values for kanamycin and for tetracycline in real sample analysis [27]. For the detection of 4-nitrophenol in tap water samples, the MXene sensor electrodes showed recoveries [6], while the PANI-Ti_3_C_2_/GCE platform showed recoveries for the detection of mercury in natural water samples [23, 27]. The recoveries of Alk-Ti3C2/N-PC/GCE and Ti3C2/MWCNTs/GCE electrodes were evaluated for the simultaneous determination of catechol and hydroquinone in industrial effluents [23]. The recoveries obtained by Gr/MXene/GCE for BPA in aqueous and dairy samples were evaluated [23]. For pharmaceutical applications, a sensor based on a boron-doped diamond nanoparticles and reduced graphene oxide modified screen printed carbon electrode for azithromycin showed a recovery in hospital effluent [142]. The selectivity of the Nafion/reduced graphene oxide-modified GCE for determination of amoxicillin in river water was satisfactory in the presence of benzylpenicillin and humic acids, but the efficiency of the sensor was somewhat decreased by the high concentration of humic acids. The recovery values satisfy the analytical acceptance criteria stated in the regulatory directives on environmental monitoring. The difference in recovery rates for the different analytes and matrices shows that calibration procedures based on the matrix and validation processes according to the kind of water are needed.

Considerable progress has been made in techniques to decrease matrix interference and preserve sensor efficiency in complex aquatic environments. The most utilised approach is the application of antifouling surface coatings. The incorporation of zwitterionic polymer coatings such as sulfobetaine methacrylate (SBMA) into MXene-based sensor systems has been reported; PSBMA@SiO2-MXene composites on glassy carbon electrodes displayed good antifouling performance for oxytetracycline detection in a protein-containing environment [5]. Inspired by the adhesion mechanisms of mussels, polydopamine coatings can be used to protect the surface of MXene from oxidative degradation and to provide active sites for subsequent functionalization, and MXene-polydopamine composites have been demonstrated to maintain sensitivity after adsorption-desorption cycles [5, 100]. Initial sensitivity was maintained during continuous operation in complex biological settings for zwitterionic and polydopamine coatings [5, 88]. Conducting polymer intercalated MXene nanosheets, notably polypyrrole, enhance the electron conductivity and also reduce biofouling via steric hindrance mechanisms [5, 89]. The sample preparation methods such as dilution, filtration, and standard addition methods are essential for matrix-matched calibration in the analysis of wastewater with high organic load [142, 143]. Nafion membrane is used as a protective barrier to prevent the interference of macromolecular interferents to the electrode surface allowing only small molecules of analyte. MXene-polymer hybrid sensors have shown better performance with Nafion coated sensors showing drift in the signal under similar conditions. MIP recognition layers on MXene substrates have artificial antibody-like specificity and low cross-reactivity for structural analogues and low intra-batch variability, which significantly reduces nonspecific binding in complex matrices [27, 66]. Advanced computational methods, such as machine-learning-assisted calibration, have been demonstrated to improve the quantification accuracy in environmental samples compared to LC-MS/MS standards [138]. Devices with internal referencing, such methylene blue-MXene-Au combinations, are ratiometric sensing devices that intrinsically adjust for signal drift produced by the matrix. Collectively, these technologies provide a pathway to the development of robust, field-deployable MXene sensors capable of reliably quantifying PPCPs in a range of aquatic habitats.

### Critical comparison of MXene sensor architectures, recognition strategies and translational gaps

A simple analysis of reported MXene sensors demonstrates that no one design could be considered as universally superior for all matrices and categories of PPCP, but the efficacy is highly dependent on the combination of MXene configuration, surface modifications, recognition processes and sample matrix [150–152]. Untainted and delaminated Ti_₃_C_₂_T_x_ has high conductivity and easily accessible surface locations, but composite structures frequently provide a better compromise between signal amplification, selectivity, and operational longevity. In particular, MXene-polymer and antifouling-coated structures are useful for extended functioning in complex aquatic settings, whereas noble-metal/MXene and MXene-MOF composites are superior when catalytic signal amplification and low detection thresholds are the critical objectives [150–152]. The current evidence suggests that sensor performance should be evaluated considering matrix compatibility and operational stability, in addition to LOD, and not just the lowest reported LOD as the main criterion. Aptamers, although very sensitive and molecularly specific, are potentially more prone to matrix-induced fouling and changes in recognition behavior, but molecularly imprinted polymers provide an attractive trade-off between selective recognition, chemical durability, and reusability. Enzyme identification is quick and sensitive, but long-term stability is still a problem. Such patterns are in accordance with the current MXene sensing studies emphasizing functionalization, MIP integration, and improved stability instead of relying solely on bare MXene [150–152].

In ideal laboratory conditions, buffered and synthetic samples, with tightly controlled ionic strength, pH, levels of interferents and composition of analytes, the limits of detection can be low . In natural wastewater and surface water, natural organic matter, humic chemicals, dissolved salts, suspended particles, competing ions, and structurally similar contaminants may affect adsorption, electron-transfer kinetics, and the availability of recognition sites. In addition, the electrochemical baseline and sensitivity may be progressively altered by oxidation of MXenes and changes in surface termination chemistry. A very low laboratory limit of detection must not thus be interpreted to mean that the field effectiveness is equal. The current literature suggests that matrix effects are poorly described, with some studies reporting recovery values, but few systematic evaluations of signal suppression in relation to NOM, ionic strength, and other matrix parameters.

Furthermore, the material analyzed is unevenly distributed among PPCP categories and environmental matrices. Antibiotics, analgesics and several hormones are extensively covered, but other PPCP categories, including numerous personal-care components, transformation products and compounds with weak and weakly described electrochemical characteristics, are still mostly untested. This overall split may also be seen in the sample environments: present work is mostly focused on wastewater and lab-spiked water, but full validation in raw surface waters, drinking-water sources, groundwaters and other types of urban and industrial water systems remains limited. The conflicting data base resulting from these findings implies that current conclusions on the effectiveness of MXene sensors are valid only for certain pharmaceutical classes and specific water matrices, not for all PPCPs and aquatic environments. Future studies should incorporate cross-matrix validation, standardized comparisons with reference methods, multi-analyte detection, extended operational range and testing at environmentally relevant concentrations rather than simply increasing the limit of detection under ideal laboratory conditions.

## Challenges and opportunities

**Fig. 17:**
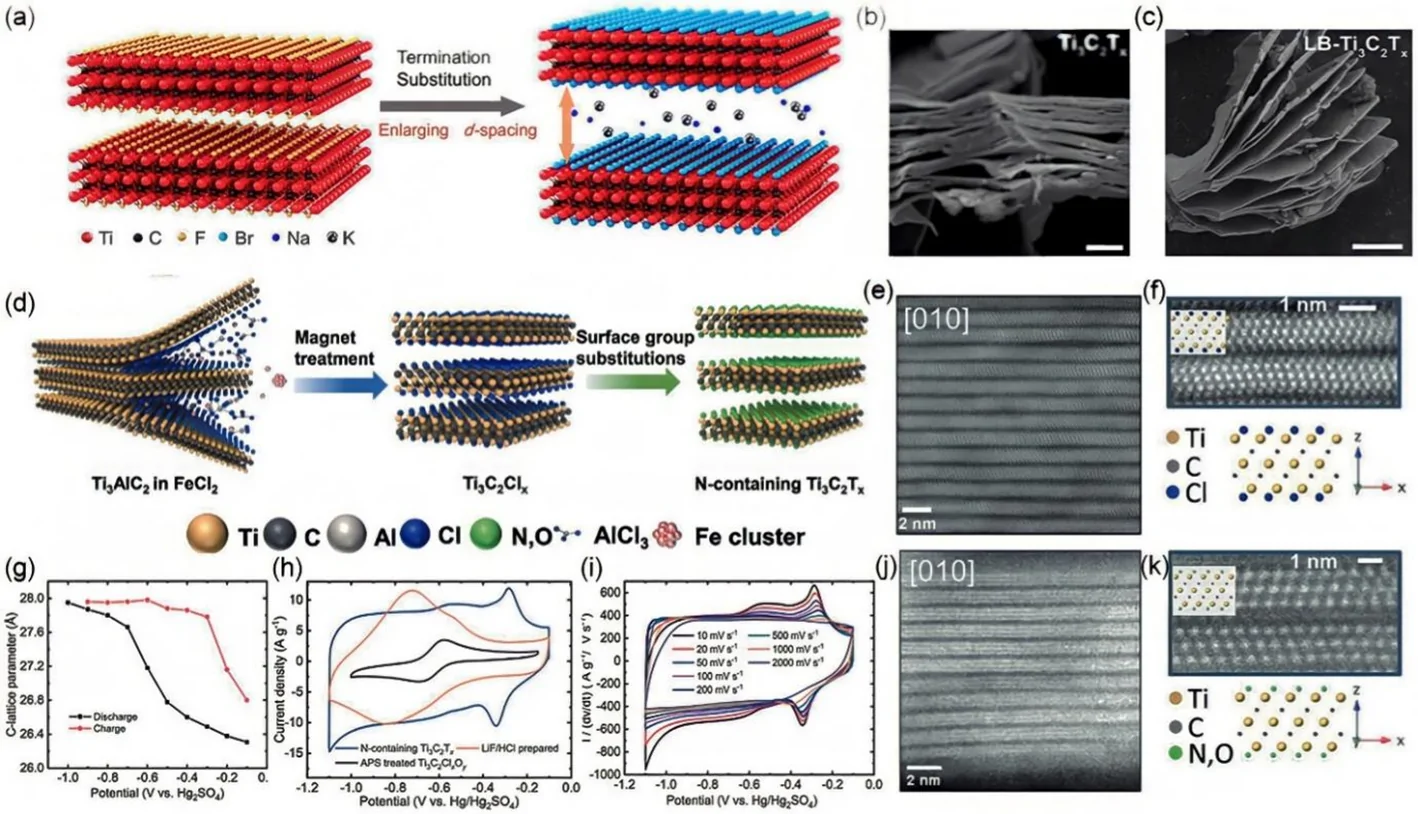
(I) **a**-**c** Structural modulation and surface functionalization of MXenes through substitution, intercalation, and defect engineering [158]. **d**-**i** schematic of Ti₃C₂ functional groups with magnetic and electroactive moieties [40]

**Fig. 18:**
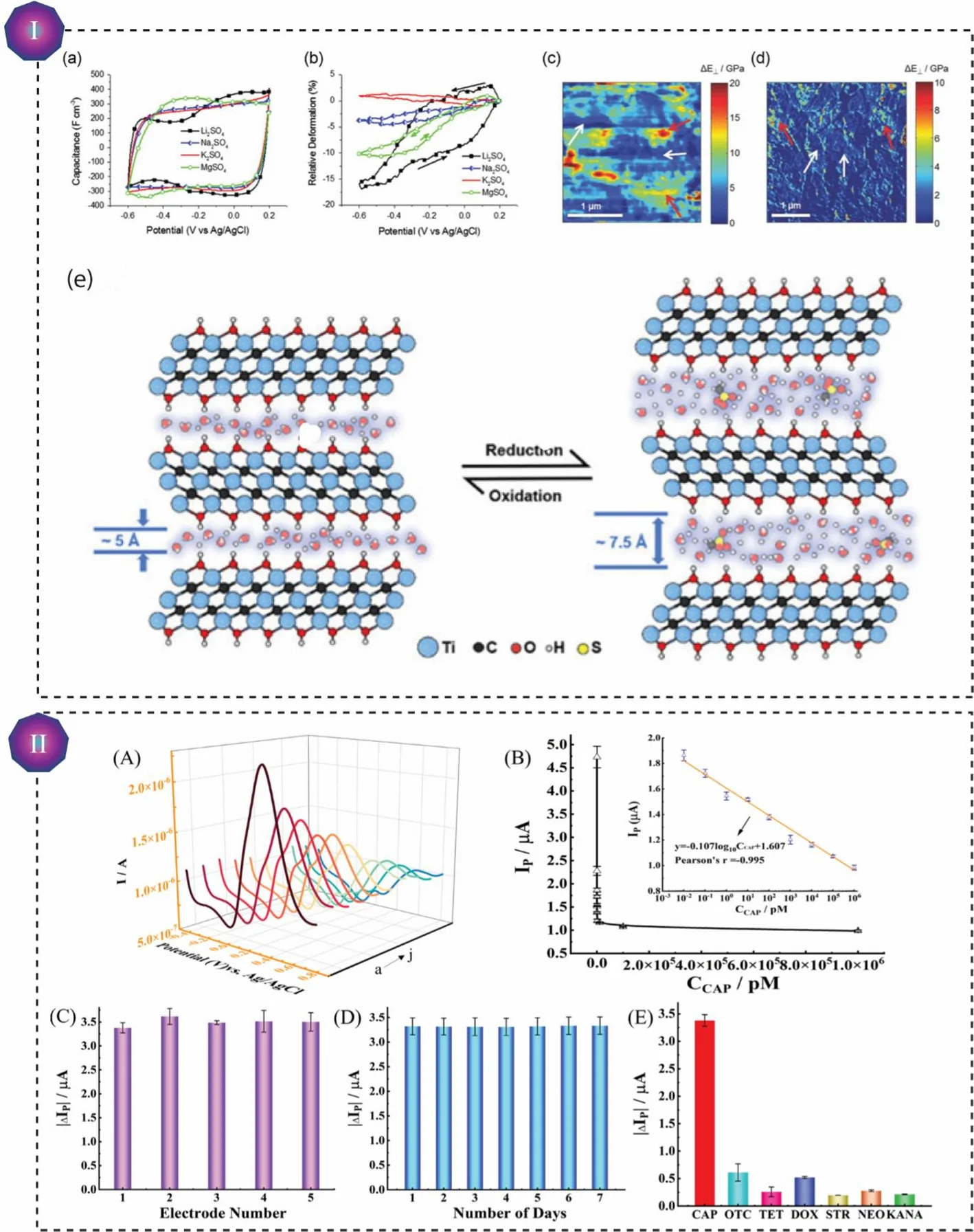
(I) Cyclic voltammetry and galvanostatic charge-discharge analysis of MXene composites at various scan rates and current densities, highlighting stable electrochemical performance [159–161]. (II) differential pulse voltammetry spectra for target analytes and calibration plot for concentration-dependent detection [72]

There are numerous key challenges to be addressed before MXene-based electrochemical sensors can become completely economically feasible, from experimental research to practical environmental monitoring applications [10]. The fundamental problem is the scalable and low-cost manufacture of high-quality MXenes. Currently, the processes often include the use of toxic chemicals, such as hydrofluoric acid, and cumbersome purification protocols, resulting in high costs and safety issues [148]. Recent improvements in fluoride-free and milder etching processes are promising for more sustainable and industrially practical synthetic operations, although additional tuning is needed to hold constant material quality and performance [23]. There are further issues such as the long-term stability and longevity of MXene sensors under environmental settings. MXenes are inherently prone to oxidation and moisture, which may degrade their conductivity and surface properties, leading to poor endurance and reliability of the sensors [149]. Research is continuing on efficient surface passivation, encapsulation and development of antifouling coatings, all with the objective to improve the robustness of MXenes without compromising on sensitivity [153]. Extensive aging studies and long-term field validation are required to build confidence in the reliability of MXene sensors for regulatory approval and practical applications [154]. From the point of view of commercialization, methods for the manufacture such as solution processing, inkjet printing and layer-by-layer assembly are researched for homogenous and repeatable sensor devices. Such devices may be used on flexible substrates and portable shapes for field applications [155]. The use of digital platforms such as Internet of Things (IoT) frameworks and machine learning algorithms might further enhance the sensing capabilities of MXenes by allowing real-time multi-parameter monitoring and advanced data analysis. Finally, effective lifecycle management of MXene-based sensors is required to avoid the anticipated environmental and health concerns associated with nanoparticle emission, metal content and disposal methods [156]. Recycling strategies, safe handling methods, and eco-friendly packaging might foster sustainable sensor commercialization [157]. Continuous cross-disciplinary collaborations and targeted innovation to solve the technological, environmental and regulatory challenges can turn MXene-based electrochemical sensors into powerful tools for ultra-trace monitoring of pharmaceuticals and personal care products in aquatic environments [15]. This will bolster their efforts to scale up action worldwide to protect the environment and public health. Extensive studies have been done on many approaches of structural modification and surface functionalization of Ti_3_C_2_T_x_ MXenes for increasing their electrochemical characteristics and stability (Figs. 17 and 18).

MXenes are generated by selective etching of A-layer atoms, e.g., Al from Ti₃AlC₂, using fluorine-based acids such as HF and LiF/HCl. During this process, the exposed MXene surface is simultaneously functionalized with a variety of termination groups, including –F, –OH, –O, and depending on the etching chemistry, –Cl. The relative abundance and distribution of these surface terminations are very sensitive to synthesis parameters like etch time, temperature, acid strength, precursor composition, and post-etch rinse procedures [140, 162–165]. Thus, the surface chemistry of MXene may be very different for different batches even when the nominal synthesis conditions are reproducible. This has a direct impact on the physicochemical and analytical performance of MXene-based materials. Surface termination composition could have a crucial role in determining the electrical properties of MXenes. Conductivity of oxygen-terminated Ti₃C₂Oₓ , but the conductivity of hydroxyl- and fluorine-rich surfaces is strongly suppressed because of increased dispersion of charge carriers and modification of electronic structure [140, 162–165]. These oscillations are of particular significance for electrochemical and resistive sensing systems, since the transfer of charge inside the MXene framework is directly linked to the generation of the signal. For piezoresistive strain sensors, the gauge factor has been found to vary between different batches fabricated under the same conditions, which has been linked to changes in the surface termination. This highlights the impact that subtle differences in surface chemistry can have on the performance of the device. Surface terminations also affect the adsorption qualities and the molecular recognition characteristics of MXenes [140, 162–165]. For gas sensing, the oxygen-terminated surfaces may exhibit stronger interaction with analytes such as NO₂. The adsorption energies of oxygen-terminated surfaces are found to be larger than those of fluorine-terminated surfaces. Accordingly, batch-to-batch fluctuations in the ratio of the –O/–F/–OH terminations might affect analyte adsorption, desorption kinetics, and charge-transfer dynamics. In operational sensing systems these oscillations may manifest as changes in baseline resistance and current, variations in the amplitude of the response, irregular baseline drift, and increased cross-sensitivity to interferents [140, 162–165]. Similar trends might be expected for electrochemical sensing, where surface functional groups impact the adsorption of analytes, interfacial charge transfer, the wettability of electrodes, and interactions with recognition elements and functional coatings. Various methods have been proposed to decrease the variation of the surface chemistry of MXenes in various batches. Thermal annealing can control the surface termination and can also partially stabilize the surface terminations. Alternative synthetic routes such as Lewis-acid molten-salt etching may improve the control over the chemical composition of the resultant MXene surface and decrease the dependence on the traditional fluorine-based etchants. However, these approaches may need additional processing steps, specific equipment requirements and higher manufacturing costs [140, 162–165]. Therefore, detailed surface analysis at the batch level is important for consistent MXene functioning. Methods like X-ray photoelectron spectroscopy (XPS) and electron energy-loss spectroscopy (EELS) may provide important information on the elemental composition, chemical states and relative amounts of surface termination groups. However, the systematic characterization of surface chemistry for each synthesized batch is still recorded inconsistently in scientific literature, which is a substantial barrier to the repeatability, comparability and eventual scalability of MXene-based sensing devices.

 Show thermodynamic metastability of MXenes, notably Ti₃C₂Tx, under ambient conditions. Oxidation happens via a water- and oxygen-assisted process, mainly at the edges and defect sites, yielding TiO₂ and amorphous carbon. Under ambient conditions conductivity may decrease over time [162, 166–169]. However, an aqueous solution in air-saturated environments may cause a decrease in conductivity . Instead, polymer encapsulation (PVA, PDMS) may limit the conductivity drop , and inert storage in argon, away from light, at low temperature can provide negligible degradation . High temperature and humidity greatly accelerate oxidation [162, 166–169]. Increasing temperature can accelerate oxidation. Losses in conductivity of unprotected MXene films may occur under elevated temperature and humidity. This degradation also leads to a large variation in the signal in sensing applications. The baseline resistance of unencapsulated MXene electrodes in wearable sweat sensors may change , making long-term monitoring difficult without protective encapsulation. The chemical environment plays an important role in the stability of MXenes [162, 166–169]. Acidic and alkaline conditions accelerate the etching of titanium atoms on the surface, and reduce the lifetime of the sensors compared with neutral conditions. Techniques such as polymer encapsulation, antioxidant doping and surface passivation have been used to improve the stability of MXenes, however they may sometimes come at the expense of reduced sensitivity and flexibility. The PVA coating may reduce the oxidation but may increase the response latency owing to the extra diffusion barrier [162, 166–169].

Short-term testing suggests positive biocompatibility, however long-term toxicological properties of MXene nanoparticles are not well understood, especially for environmental release scenarios [170–172]. Ti₃C₂Tx has low acute toxicity in fibroblast and keratinocyte cell lines during short-term exposure , but oxidative stress and mitochondrial damage are observed after longer exposure at elevated concentrations . Data on aquatic organisms are lacking, but early studies suggest Ti₃C₂Tx nanosheets may bioaccumulate in the gut tissues of Daphnia magna [170–172]. MXenes may degrade into TiO₂ nanoparticles and soluble Ti⁴⁺ in soil and water over a period of weeks to months, however the rate of degradation is dependent on parameters such as pH, redox potential and microbial activity. Moreover, there are no defined procedures for the disposal of MXenes and emission threshold limits yet, and current risk assessment is mostly based on TiO₂ and graphene information, which may not be adequate to represent the particular reactivity of MXenes. Therefore, life cycle assessment (LCA) studies, standardized ecotoxicity approaches and the development of biodegradable MXene composites are necessary for the reduction of risks [170–172].

The high cost and energy-intensive production of MXene limits its competitiveness against well-established alternatives, such as carbon black, graphene, and metal oxides [140, 162, 163]. Scalability of MXene is currently relatively limited, with laboratory production being limited to small quantities, whereas carbon black and metal oxides may be produced at larger industrial scales [140, 162, 163]. There are several synthetic restrictions that contribute to high costs of MXene manufacture. The use of HF-based etching for production requires corrosion-resistant reactors, disposal of hazardous waste, and many purification steps, which increase manufacturing costs . Furthermore, the delamination efficiencies can be limited, which leads to substantial material losses during the washing and centrifugation steps [140, 162, 163]. Currently, the total global production of MXene is estimated to be limited Cost minimization strategies include fluorine-free etching methods such as molten-salt and electrochemical processes, the use of continuous-flow reactors for scalable delamination, and the development of hybrid composites such as MXene/carbon black systems, which reduce the amount of MXene required while maintaining the desired sensing efficacy [140, 162, 163].

The use of MXene-based sensors in the environmental field is faced with a significant issue of sustainability: a sensor that detects pollutants should not cause secondary contamination. MXenes have enticing analytical characteristics, but their environmental use requires an exhaustive study of material emissions over their lifecycle: production, operation, degradation, maintenance, and disposal [173]. Mechanical wear, surface abrasion, and delamination and degradation of polymeric and other protective layers may potentially release MXene-containing particles into aquatic habitats, and oxidation may also alter intrinsic MXene structure and impact its interactions in the environment. MXenes released into the environment are poorly characterized in terms of long-term fate, avenues of transformation, bioavailability and ecological consequences, particularly under realistic aquatic exposure scenarios [174]. However, the present knowledge is not sufficient to infer that the environmental risk of an MXene sensor is low only because the active material is immobilized during normal operation. Crucially, the risk evaluation must encompass the whole sensor, not just the unmodified MXene, because composites may include polymers, conductive additives, precious metals, and other constituents that might increase environmental damage [175]. Looking ahead, sensor innovation should adopt a lifecycle perspective in design, including robust immobilization and encapsulation of MXene, resistance to mechanical and chemical degradation, minimizing active-material loading, recovery and recycling of the sensing component, and controlled disposal at the end of its life. Environmental safety evaluations should be performed in parallel with analytical validation and should include metrics for material release, degradation and transformation analyses, ecotoxicological assessments utilizing relevant aquatic species and studies of repeated-use and disposal settings [176, 177]. This approach would allow us to examine MXene-based environmental sensing not only in terms of sensitivity and selectivity but also in terms of the potential to achieve a positive balance between the benefits of pollutant monitoring and its own environmental effect.

## Discussion

This study is an important addition to the intersection between environmental nanotechnology and analytical electrochemistry. The concern is based on the rising threat of PPCPs to the environment. PPCPs represent a significant class of contaminants that persist in aquatic environments even at low concentrations [178, 179]. These compounds are persistent, likely to bioaccumulate and are endocrine-disruptive therefore extremely sensitive detection equipment is a necessity. It positions itself in the global environmental discussion, presenting MXene-based sensing technologies as novel analytical tools for connecting environmental monitoring and public health surveillance [180]. The research provides compelling evidence that MXenes, 2D transition metal carbides and nitrides, are a big advance in electrochemical sensing. Their excellent electrochemical reactivity to pharmaceutical residues [181, 182] is rooted in the combination of their metallic conductivity with programmable surface chemistries (-O, -OH, -F) and hydrophilicity. Low limits of detection have been reported under ideal circumstances. MXenes exhibit higher charge-transfer kinetics and surface adsorption characteristics compared to graphene and MoS_2_. The scientists define these materials as translationally good to classic 2D systems, compatible with aqueous matrices and resilient to common environmental interferents such as humic acids and heavy metals [183–185]. We cover major developments in surface functionalization, such as polydopamine, zwitterionic coatings and redox-stable polymer encapsulation, that minimize MXene oxidation and increase selectivity. These modifications may improve longevity and antifouling properties, therefore ensuring operational sensitivity during continuous usage [186, 187]. Coupling of materials chemistry with functional design is an excellent example of an interdisciplinary challenge of MXene sensor development. The essay gives a wonderful contribution to the field with the analysis of the bio-inspired and biomimetic way of sensor design. Purposeful biomimetic engineering strategies rely on the inclusion of natural recognition processes such as peptide-functionalization for mimicking fish olfactory receptors, molecular imprinting for imitating antibody-like selectivity and enzyme immobilization on MXene substrates [188, 189]. The systems emulate biological recognition with enhanced structural stability and reusability with low intra-batch variability and a decrease in cross-reactivity. Bio-inspired design is not confined to replicating chemical features, with MXene hydrogels and flexible composites demonstrating tissue-like traits, such as the integration of electrical conductivity with mechanical flexibility and self-healing capabilities [190]. The Young’s modulus of these hydrogels is equivalent to that of soft biological tissues, making them intriguing materials for wearable and implantable biosensors. One example of the intersection of ecological sensing and biomedical engineering, the authors cite, is the introduction of bioinspired materials to environmental monitoring systems. MXene-based electrochemical and OECT systems have been investigated for the detection of antibiotics and other medicinal compounds [191]. In addition, the high repeatability , quick reaction time and operational stability ( in physiological and aquatic settings) further illustrate the maturity of MXene technologies for real-world application. The essay also focuses equally on clinical and environmental validation. An example of the translational maturity of the European multi-city wastewater epidemiology project is provided, where agreement with LC-MS/MS measurements was assessed . The findings show that the use of MXene sensors as analytical and epidemiological instruments for real-time pharmaceutical monitoring in urban wastewater is feasible [192]. This validation goes from laboratory proof-of-concept to regulatory application and smart city environmental monitoring. MXenes may provide a bridge between environmental monitoring, clinical diagnostics and public health surveillance to be both ambitious and ingenious. MXene-based sensors are a convergence technology of materials science, bioengineering, and data analytics, which allow for real-time ultra-trace detection of PPCPs in diverse aquatic matrices for tackling a worldwide environmental problem [193–196]. This adaptability of MXene-based structures makes them capable of detecting new contaminants, monitoring diseases and diagnosing biomarkers. The study is fairly extensive, integrating published findings with conceptual synthesis. Future revisions may benefit from a more extensive analysis assessing inter-study variance and effect sizes for sensor combinations. Moreover, a more in-depth study of the long-term environmental persistence and toxicity of MXenes can address concerns of secondary contamination [197–200]. This will also enhance the translational and regulatory rigor of the study. This work presents a holistic, future-oriented and multidisciplinary review of MXene-based electrochemical and bio-inspired sensing devices. MXenes are regarded as breakthrough nanomaterials that will revolutionize environmental analytical chemistry and inaugurate an age of intelligent real-time pollution monitoring [201–204]. The study covers cutting-edge research and a clear path for future exploration with a focus on sustainability, integration and translational application. It is a tremendous development in the fields of research on nanomaterials, environmental sensing and bioanalytical innovation.

## Conclusion

The MXene-based electrochemical and bio-inspired technologies possess the potential to make a significant leap for the sensitive, selective and rapid detection of PPCPs in complex aquatic settings. They are particularly promising due to their unique physicochemical properties such as high conductivity, large surface area, flexible surface chemistry and different functionalization techniques. These properties allow us to handle the analytical challenges of PPCP contaminants at the ultra-trace level. This study focuses on the basic building blocks of MXene nanomaterials, the latest advances in sensor design, incorporation of bio-inspired recognition features, and analytical performance from an environmental standpoint. Despite remarkable gains, there are still fundamental challenges that restrict the full translation of MXene sensor technology from lab prototypes to field-deployable devices. Major issues include scalable and secure synthesis methods in environmentally friendly processes, reproducible synthesis techniques in ecologically friendly procedures. For enabling robust real-time environmental monitoring, there is a need for better long-term stability of sensors against oxidation and biofouling, better multiplexing capabilities and linkage with a smart data analytics framework. Also, the legal and sustainability aspects, such as lifetime management and circular economy integration, will be considered for responsible commercial implementation. Accelerated interdisciplinary research in materials science, electrochemistry, bioengineering and environmental science will be needed to fully unlock the potential of MXenes for PPCP sensing. Novel approaches such as in situ sensor renewal, self-sustaining sensing via energy harvesting, and artificial intelligence-enabled wireless sensor networks have great potential to alter the environmental monitoring sector. Further research and collaboration will turn MXene-based sensors into vital tools in worldwide efforts to safeguard aquatic ecosystems, public health and water quality in a period of rising pollution and shrinking resources.

## Data Availability

No datasets were generated or analysed during the current study.
